# Neratinib protects pancreatic beta cells in diabetes

**DOI:** 10.1038/s41467-019-12880-5

**Published:** 2019-11-01

**Authors:** Amin Ardestani, Sijia Li, Karthika Annamalai, Blaz Lupse, Shirin Geravandi, Aleksandra Dobrowolski, Shan Yu, Siying Zhu, Tyler D. Baguley, Murali Surakattula, Janina Oetjen, Lena Hauberg-Lotte, Raquel Herranz, Sushil Awal, Delsi Altenhofen, Van Nguyen-Tran, Sean Joseph, Peter G. Schultz, Arnab K. Chatterjee, Nikki Rogers, Matthew S. Tremblay, Weijun Shen, Kathrin Maedler

**Affiliations:** 1 Centre for Biomolecular Interactions Bremen, University of Bremen, Bremen, Germany; 2 Calibr at Scripps Research, La Jolla, CA USA; 3 Center for Industrial Mathematics, University of Bremen, Bremen, Germany; 4 MALDI Imaging Lab, University of Bremen, Bremen, Germany

**Keywords:** Mechanisms of disease, Type 2 diabetes

## Abstract

The loss of functional insulin-producing β-cells is a hallmark of diabetes. Mammalian sterile 20-like kinase 1 (MST1) is a key regulator of pancreatic β-cell death and dysfunction; its deficiency restores functional β-cells and normoglycemia. The identification of MST1 inhibitors represents a promising approach for a β-cell-protective diabetes therapy. Here, we identify neratinib, an FDA-approved drug targeting HER2/EGFR dual kinases, as a potent MST1 inhibitor, which improves β-cell survival under multiple diabetogenic conditions in human islets and INS-1E cells. In a pre-clinical study, neratinib attenuates hyperglycemia and improves β-cell function, survival and β-cell mass in type 1 (streptozotocin) and type 2 (obese Lepr^db/db^) diabetic mouse models. In summary, neratinib is a previously unrecognized inhibitor of MST1 and represents a potential β-cell-protective drug with proof-of-concept in vitro in human islets and in vivo in rodent models of both type 1 and type 2 diabetes.

## Abstract (web summary)

Type 1 as well as type 2 diabetes are characterized by a loss of insulin-producing β-cells. Here the authors show that the FDA-approved drug neratinib has beneficial effects on β-cell survival, insulin secretion, and glycemic control in mouse models of diabetes.

## Introduction

Loss of function and/or mass of pancreatic β-cells is a critical pathogenic hallmark of both type 1 and 2 diabetes (T1D/T2D)^1–5^. Pancreatic β-cell apoptosis contributes to the loss of insulin-producing β-cells in diabetes, rapidly induced by the activation of the immune system in T1D and slowly progressing in T2D^1–4,6–11^. In addition, β-cell dedifferentiation^12–14^ and failure of adaptive expansion due to impaired proliferation^15,16^ are other proposed mechanisms for the loss of functional β-cell mass in diabetes. The mechanisms of β-cell failure are complex; multiple triggering factors have been identified, which initiate signaling cascades that affect the expression of apoptotic genes. The development of novel agents that can selectively block β-cell apoptosis together with the restoration of β-cell function with safety profiles commensurate with the treatment of chronic disease is urgently needed. Current therapies for the treatment of diabetes are directed toward alleviating only the symptoms, i.e., the normalization of glycemia through enhanced insulin secretion from the remaining β-cells, and the improvement of insulin sensitivity in T2D, and through tightly controlled exogenous insulin therapy in T1D. None of the currently used antidiabetic agents target the maintenance of endogenous β-cell mass, although it has been demonstrated that even a small amount of preserved endogenous insulin secretory function has great clinical benefits^17^.

In our previous work, we identified mammalian sterile 20-like kinase 1 (MST1, also known as STK4, KRS2) as a critical regulator of pancreatic β-cell death and dysfunction^11^. MST1 is a ubiquitously expressed serine/threonine kinase, the major upstream signaling kinase in the Hippo pathway, involved in multiple cellular processes, such as morphogenesis, proliferation, stress response, and apoptosis^18,19^. MST1 is a direct target as well as an activator of caspases, forming a feed-forward loop that drives the apoptotic signaling pathway^20,21^. MST1 promotes cell death through regulation of multiple downstream targets, such as LATS1/2, histone H2B, FOXO family members, the intrinsic mitochondrial proapoptotic pathway, stress kinase c-Jun-N-terminal kinase (JNK), and caspase-3 activation^19,22,23^. MST1 is strongly activated in β-cells under diabetogenic conditions and its activity correlates with β-cell apoptosis and degradation of PDX1^11,24^, a β-cell transcription factor highly important for β-cell identity, survival, and function^25^. MST1 deficiency markedly restores β-cell function and survival and leads to protection of β-cell mass and normoglycemia in mouse models of diabetes^11^. The identification and elaboration of MST1 inhibitors represents a promising approach to β-cell-protective drugs for the treatment of diabetes.

Several series of MST1 inhibitors have been reported, demonstrating the feasibility of generating potent, selective small-molecule inhibitors^26–29^. Through a biochemical MST1 inhibition screen across a highly privileged collection of 641 drug-like kinase inhibitors, we identified neratinib as a potent MST1 inhibitor. Neratinib is a covalent, irreversible ATP-competitive dual inhibitor of HER2/EGFR. The epidermal growth factor receptor (EGFR, also named ErbB-1/HER1) and human epidermal growth factor receptor 2 (HER2, also named ErbB-2) are tyrosine kinases of the ErbB family and involved in organ development and growth, as well as in the pathogenesis of various tumors^30^. FDA approved for the treatment of breast cancer^31–33^, neratinib is also in clinical trials for lung, colorectal, and bladder cancers. Via its acrylamide moiety, neratinib forms a covalent interaction with the conserved cysteine residue (Cys-773 in EGFR and Cys-805 in HER2), resulting in tight engagement of the ATP-binding site and robust inhibition of the activation of the EGFR signaling pathway and cell proliferation^34^. However, this conserved cysteine is not present in MST1.

In this study, we report neratinib as a β-cell-protective kinase inhibitor in proof-of-concept experiments in a widely used β-cell line, in human islets, as well as in both T1D and T2D rodent models. Specifically, the goal of this work was to evaluate neratinib’s efficacy to prevent apoptosis in human islets and to restore normoglycemia in the streptozotocin (STZ)-induced and in the obese Lepr^db/db^ diabetic mouse models.

## Results

### Neratinib was identified as MST1 inhibitor

**Fig. 1:**
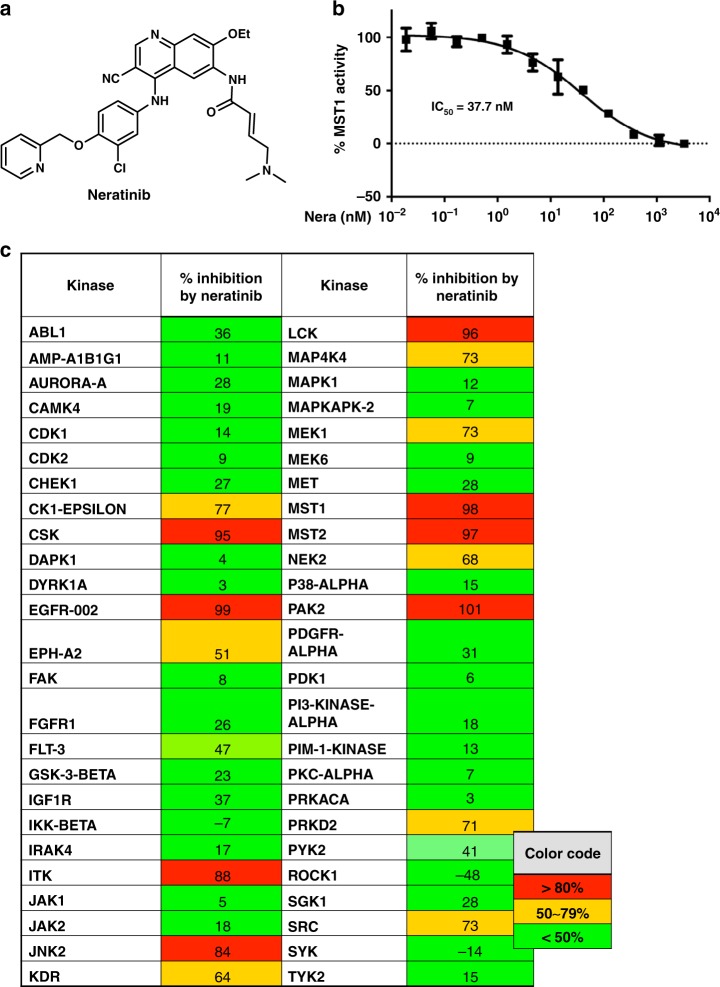
Neratinib, a kinase inhibitor with MST1 efficacy. **a** Chemical structure of neratinb. **b** Biochemical dose response confirmation of MST1 inhibition. Data show means ± SEM from three independent experiments (*n* = 3). **c** Kinase profiling showing % kinase inhibition at 10 µM neratinib with a panel of 50 kinases, means of duplicates are shown. Source data for (**b**) are provided as a Source Data file

To identify novel MST1 inhibitors, we developed a high-throughput LanthaScreen Eu kinase binding assay platform in 1536-well format. A focused library of 641 annotated kinase inhibitor compounds was screened at 5 concentrations (1, 0.2, 0.04, 0.008, and 0.0016 μM). With staurosporine as the positive control, we chose hits with ≥75% inhibition of binding at 1 μM and ≥50% inhibition at 0.2 μM compared with staurosporine, which exhibited 100% inhibition at 1 μM. A total of 39 hits were selected for 9-point dose response confirmation in triplicate, starting at 1 μM followed by 1:5 serial dilution. Neratinib was identified as a potent inhibitor of MST1 (IC_50_ = 37.7 nM for the binding assay) (Fig. 1a, b).

We then re-profiled neratinib in a representative panel of 50 serine, threonine, and tyrosine kinases, which revealed inhibition of 16 serine/threonine and tyrosine kinases with >50% of inhibition at 10 µM, including 98% MST1 inhibition by neratinib (Fig. 1c). Following the above results, we expanded the kinase assay panel to 250 kinases, which revealed inhibition of 59 serine/threonine and tyrosine kinases with 50% of inhibition at 3 µM, reconfirming 97% MST1 inhibition by neratinib (IC_50_ = 91.4 nM for the activity-based assay; Supplementary Fig. 1a). We further evaluated these targets in dose–response experiments to 38 kinases in the panel, including EGFR, MAP4K4, MST1, and MST2, which showed consistent potency of neratinib on these four kinases, as well as potent inhibition on LOK, MAP4K5, and YES (Supplementary Fig. 1b). Further expanded dose–response experiments revealed neratinib’s IC_50_ values of 1.79 nM for the well-known EGFR inhibition, 33.48 nM for MAP4K4, and 87.81 nM for MST2, indicating only a limited activity of neratinib toward MST2 (Supplementary Fig. 2).

### Neratinib blocks MST1 activation and apoptosis in β-cells

**Fig. 2:**
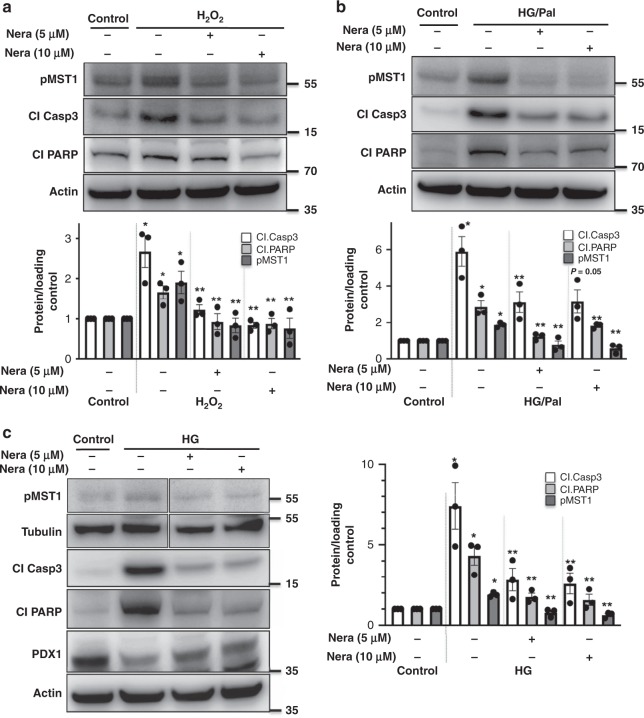
Neratinib blocks MST1 activation and apoptosis in INS-1E β-cells. INS-1E cells were exposed to diabetogenic conditions (**a** H_2_O_2_, **b**, **c** 22.2 mM glucose or the mixture of 22.2 mM glucose and 0.5 mM palmitate (HG/Palm)) ± neratinib for 72 h. Phospho-MST1 (pMST1; pThr183), caspase-3 and PARP cleavage, PDX1, tubulin, and actin were analyzed by western blotting. Representative Western blots and pooled quantitative densitometry analysis are shown from three independent cell line experiments (*n* = 3). Results shown are means ± SEM. *p < 0.05 H_2_O_2_ or HG or HG/Pal to control, **p < 0.05 neratinib to vehicle-treated β-cells under the same diabetogenic conditions; all by Student’s *t* tests. Source data are provided as a Source Data file

To identify whether neratinib can inhibit MST1 activation and restore β-cell survival under chronic diabetogenic conditions, we exposed the INS-1E cells to various stress conditions in vitro (oxidative stress: H_2_O_2_, increasing glucose concentrations alone: glucotoxicity or in combination with palmitic acid: glucolipotoxicity, and ER stress: thapsigargin). As shown previously^11^, MST1 was highly upregulated by all diabetic conditions upon chronic exposure, shown by its autophosphorylation (pMST1–T183; Fig. 2). In contrast, neratinib potently inhibited H_2_O_2_- and high glucose/palmitate-induced MST1 activation and apoptosis as represented by caspase-3 and PARP cleavage in β-cells (Fig. 2a–c). Also, neratinib restored PDX1 expression in β-cells, which was reduced by elevated glucose concentrations (Fig. 2c).

Caspase-3 activation induced by the ER stressor thapsigargin was dose-dependently abolished by neratinib, as determined by the NucView 488 caspase-3 assay (Supplementary Fig. 3a) confirming our previous data showing MST1 and caspase-3 activation by thapsigargin in β-cells, and the prevention of thapsigargin-induced apoptosis by caspase-3 inhibition^11^. Similarly, caspase-3 activation induced by the complex mixture of inflammatory cytokines (TNFα/IFNγ) and high glucose (33 mM; Supplementary Fig. 3b) as well as lipooligosaccharide (LPS)-induced expression of inflammatory cytokines TNFα, IL-1β, and IL-6 was largely inhibited by neratinib (Supplementary Fig. 3c). Neratinib treatment showed no evidence of interference on basal cell viability as determined by steady-state ATP concentrations in INS-1E β-cells at all tested concentrations (Supplementary Fig. 3d).

### Neratinib blocks MST1 activation and apoptosis in islets

**Fig. 3:**
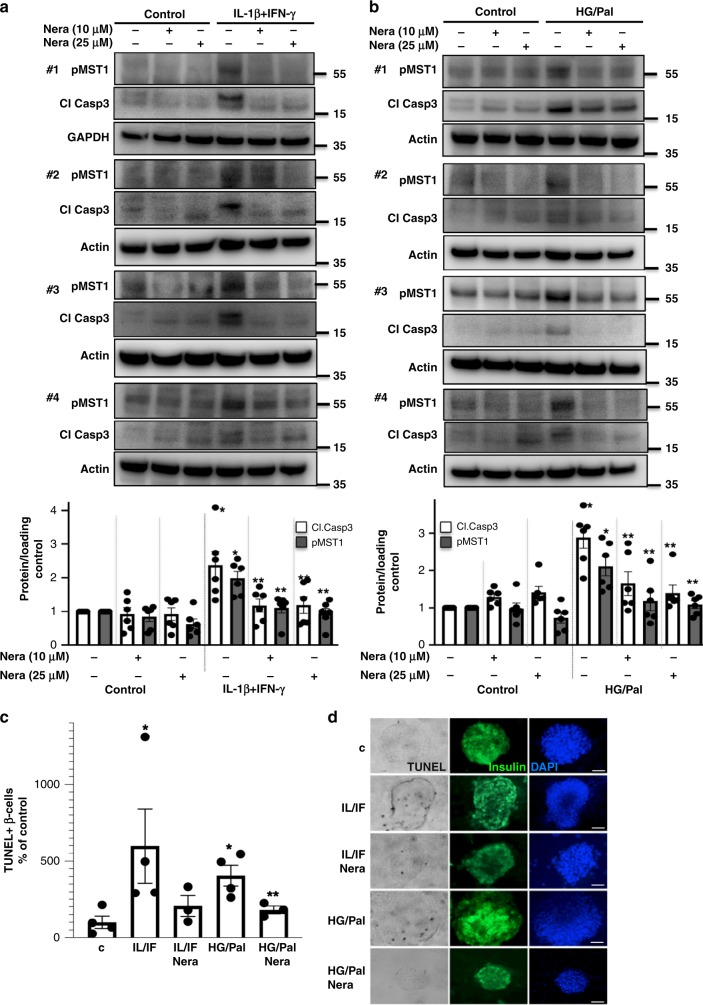
Neratinib blocks MST1 activation and apoptosis in human islets. Human islets were exposed to diabetogenic conditions (**a**, **c**, **d** IL-1β/IFNγ, **b**–**d** mixture of 22.2 mM glucose and 0.5 mM palmitate (HG/Palm)) ± neratinib for 72 h. **a**, **b** Phospho-MST1 (pMST1; pThr183), caspase-3 cleavage, and GAPDH or actin were analyzed by western blotting. Representative Western blots of four different human islet donors (**a**, **b**; upper panels) and pooled quantitative densitometry analysis (**a**, **b**; lower panels) of six different human islet donors are shown (*n* = 6). **c**, **d** β-cell apoptosis analyzed by triple staining of TUNEL (black nuclei), insulin (green), and dapi (blue). Scale bar, 100 μm. An average number of 40,420 insulin-positive β-cell per condition was counted in 3–4 independent experiments from 3 to 4 different human islet donors (*n* = 3–4). Results shown are means ± SEM. *p < 0.05 IL/IF or HG/Pal to control, **p < 0.05 neratinib to vehicle-treated islets under the same diabetogenic conditions; all by Student’s *t* tests. Source data are provided as a Source Data file

**Fig. 4:**
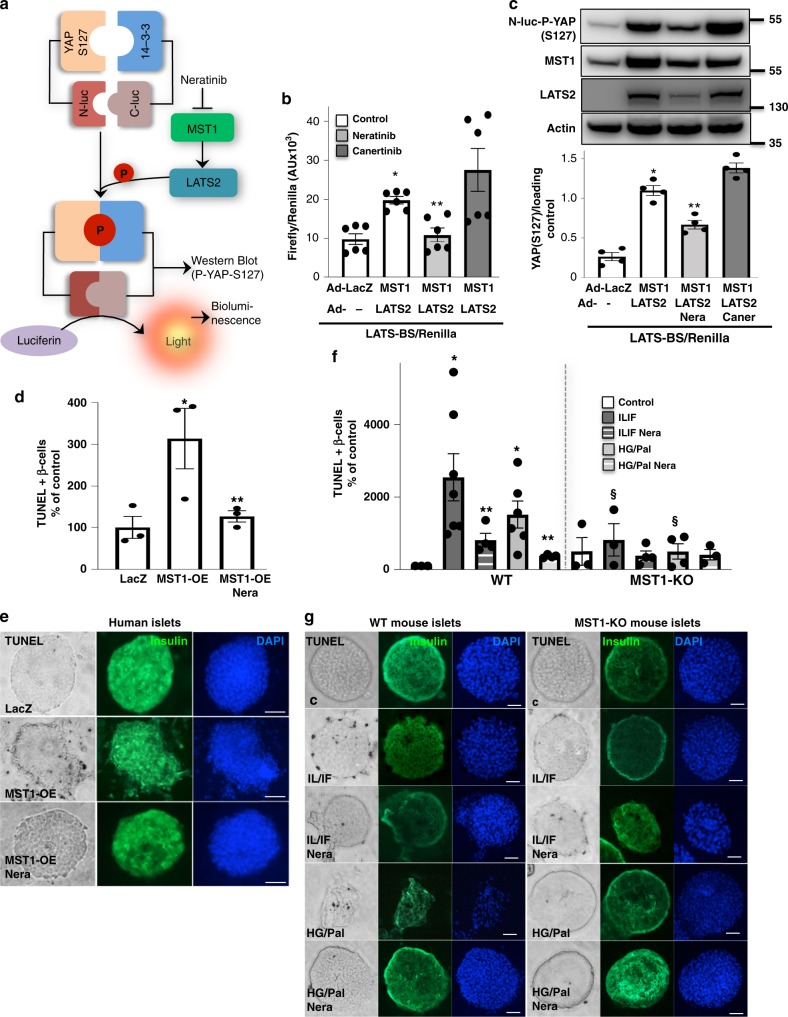
Neratinib blocks MST1 signaling and MST1-induced β-cell apoptosis. **a** Domain structure and mechanism of action for the LATS-BS. At control condition, there is no interaction between YAP and 14-3-3 showing minimal bioluminescence activity for LATS-BS (N-luc-YAP15-S127 and C-luc-14-3-3)^35^. Upon LATS activation induced by MST1, LATS-dependent phosphorylation of YAP15-S127 (analyzed by Western blotting in (**c**)) leads to 14-3-3 binding, luciferase complementation, and high biosensor signal corresponding to higher LATS activity (analyzed by bioluminescence in (**b**)). **b**, **c** Adenoviral overexpression of MST1/LATS2 or LacZ (control) in INS-1E cells, which had been transfected with the firefly luciferase reporter plasmids N-luc-YAP15-S127 and C-luc-14-3-3 as well as pRL-Renilla luciferase vector control 24 h before 10 μM neratinib or canertinib was added for the last 24 h. Downstream YAP-S127 phosphorylation was determined by luciferase activity (normalized to the Renilla signal (**b**)). Western blotting for YAP-127 phospho-specific antibody (**c**); successful transfection was confirmed by LATS2 and MST1 analysis, and actin was used as housekeeping control. Data are means from six independent culture dishes (*n* = 6; **b**) or four independent cell line experiments (*n* = 4; **c**) ± SEM. Representative Western blot is shown. **d**, **e** Human islets were infected with Ad-LacZ (control) or Ad-MST1 adenoviruses and exposed to 10 μM neratinib for 48 h. **f**, **g** Isolated islets from MST1-KO mice and their WT littermates were recovered after isolation overnight and exposed to diabetogenic conditions (IL-1β/IFNγ or the mixture of 22.2 mM glucose and 0.5 mM palmitate (HG/Pal)) ± 10 μM neratinib for 72 h. **d**–**g** β-cell apoptosis was analyzed by triple staining of TUNEL (black nuclei), insulin (green), and dapi (blue). Scale bar, 100 μm. **d** An average number of 30,700 insulin-positive β-cell per condition was counted in three independent experiments from three different donors (*n* = 3) and **f** of 5942 insulin-positive β-cells per condition from three to seven mice/condition (*n* = 3–7). Results shown are means ± SEM. *p < 0.05 MST-OE, or IL/IF or HG/Pal to Lac-Z or control, **p < 0.05 neratinib treated with MST1-OE or IL/IF or HG/Pal-treated islets to vehicle-treated islets under the same conditions; ^§^*p* < 0.05 MST1-KO to WT islets under the same diabetogenic conditions; all by Student’s *t* tests. Source data are provided as a Source Data file

The efficacy of neratinib to restore β-cell survival under multiple diabetogenic conditions was confirmed in six independent experiments by using human islet preparations from six different organ donors. Human islets were plated in a monolayer-like culture, and due to the complexity of the islet tissue culture, we also tested the higher concentration of 25 µM neratinib, which did not result in any detectable toxicity at basal control levels. Again, neratinib potently and significantly inhibited pro-inflammatory cytokine- as well as high glucose/palmitate-induced MST1 activation and caspase-3 activation in human islets (Fig. 3a, b). Further analysis of TUNEL/insulin co-positivity in isolated human (Fig. 3c, d) as well as in mouse islets (Fig. 4f, g) confirmed the anti-apoptotic action of neratinib indicating its β-cell-specific protective effect against diabetogenic condition-induced apoptosis in both primary human and mouse isolated islets.

### Neratinib blocks MST1 signaling and β-cell apoptosis

Further analyses in INS-1E β-cells (Fig. 4a–c), human (Fig. 4d, e), and mouse islets (Fig. 4f, g) confirmed that the protective effect of neratinib on β-cell apoptosis was dependent on MST1. As we observed a parallel restoration of β-cell survival and MST1 inhibition, we aimed to identify whether neratinib can specifically interfere with MST1 downstream signaling and block MST1-induced apoptosis.

Recently, a highly sensitive and reproducible bioluminescence-based biosensor (LATS-BS) that monitors the specific activity of MST1 and its downstream substrate LATS kinase in vitro in real time was developed^35^. Both MST1 and LATS2 are core kinases of Hippo signaling pathway, which act together to induce β-cell apoptosis^36^, and the specific MST1–LATS2 signaling activation can therefore be analyzed by this assay. LATS1/2 kinases phosphorylate their own established target Hippo transcriptional coactivator yes-associated protein (YAP) on S127 that exposes the docking site for binding of 14-3-3 proteins and leads to YAP cytoplasmic sequestration. Therefore a LATS-BS construct has been generated with fusion of YAP fragment and 14-3-3 with N-terminal and C-terminal firefly luciferase fragments (N-luc and C-luc), respectively that assesses LATS kinase activity by measuring the interaction between pS127-YAP and 14-3-3^35^ in a MST1–LATS2-phosphorylation-dependent manner (Fig. 4a). Adenoviral overexpression of MST1/LATS2 in YAP-deficient INS-1E^37^ β-cells transfected with LATS-BS induced strong bioluminescence-based induction of luciferase activity (represents YAP-14-3-3 final interaction; Fig. 4b) as well as YAP-S127 phosphorylation as determined by YAP-S127 phospho-specific antibody (Fig. 4c), both which was strongly inhibited by neratinib indicating its potent inhibitory action against MST1–LATS2 signaling, while canertinib, a related acrylamide-based covalent EGFR inhibitor with a similar structure to neratinib but without MST family inhibitory activity^38^ added at the same conditions had no inhibitory effect.

Consistent with our previous observations that MST1 overexpression alone was sufficient to induce β-cell apoptosis^11^, adenoviral overexpression of MST1 induced a dramatic induction of β-cell apoptosis in isolated human islets, which was significantly blocked by neratinib (Fig. 4d, e and Supplementary Fig. 4) suggesting a direct interference of neratinib with proapoptotic MST1 or its downstream signaling. To see whether MST1 is the true target of neratinib in the context of β-cell protection from apoptosis, we evaluated its effect in isolated islets from global MST1-knockout (MST1-KO) mice and their wild-type (WT) littermates. While pro-inflammatory cytokines and glucolipotoxicity highly induced apoptosis in WT mice, their harmful effect on β-cell survival was almost gone in islets isolated from MST1-KO mice (Fig. 4f, g), consistent with our previous observation^11^. Similarly, neratinib reduced cytokine- and glucolipotoxicity-induced apoptosis in WT islets, but had no additive effect in MST1-KO mice, assuming that MST1 inhibition by neratinib is sufficient to restore β-cell survival. Neratinib had no significant effect in MST1-KO islets, neither on cytokine- nor in glucolipotoxicity-induced apoptosis; these results show that neratinib specifically blocks MST1 signaling and MST1-mediated β-cell apoptosis in islets under diabetogenic conditions.

### Neratinib but not canertinib restores β-cell survival

**Fig. 5:**
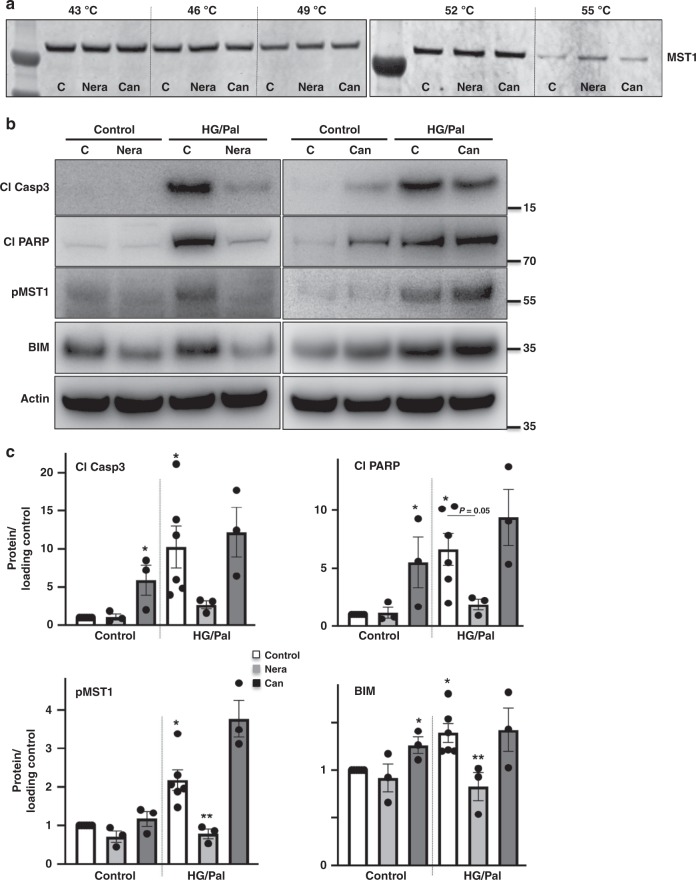
Neratinib but not canertinib binds to MST1 and restores β-cell survival through MST1 inhibition. **a** CETSA assay was performed in INS-1E cells pretreated with 5 μM neratinib, canertinib, or vehicle control, followed by heating to denature and precipitate protein at different temperatures. The stabilized MST1 protein in the soluble fraction of the cell lysate was detected by using western blotting. **b**, **c** INS1-E cells were exposed to diabetogenic conditions (22.2 mM glucose and 0.5 mM palmitate (HG/Pal)) ± 10 μM neratinib or canertinib for 24 h. Phospho-MST1 (pMST1; pThr183), caspase-3 and PARP cleavage, and BIM and actin were analyzed by western blotting. Representative Western blots (**b**) and pooled quantitative densitometry analysis (**c**) are shown from 3–6 independent culture dishes (*n* = 3–6). Results shown are means ± SEM. *p < 0.05 HG/Pal to control, **p < 0.05 neratinib to vehicle + HG/Pal-treated β-cells under the same conditions; all by Student’s *t* tests. Source data are provided as a Source Data file

To provide further incisive target validation data for MST1, cellular target engagement as well as functional studies were performed by using neratinib and a closely related EGFR inhibitor canertinib (a.k.a. CI-1033) that lacks MST1 inhibitory activity as a target control. Indeed, canertinib showed potent EGFR inhibition (IC_50_ value of 0.21 nM), but no appreciable inhibition of MST1 or MST2 at concentrations up to 10 μM in our biochemical kinase inhibition assays (Supplementary Fig. 5). Cellular target engagement assays such as the cellular thermal shift assay (CETSA) determine direct interactions between a drug and its protein target, based on drug- or ligand-induced thermal stabilization of target proteins in intact cells^39^. In this case, CETSA has been applied to assess the direct interaction of neratinib or canertinib with MST1 in INS1-E cells. Consistent with their biochemical kinase inhibition profile, a specific binding of neratinib to MST1 in live cells is suggested by MST1 stabilization at 55 °C, while its degradation occurred at the same condition with canertinib or vehicle control (Fig. 5a).

We then compared the effect of neratinib and canertinib on MST1 activation, its downstream apoptotic target, and β-cell apoptosis in metabolically stressed β-cells. While neratinib, consistently with presented data (Figs. 2 and 3), strongly counteracted stress-induced MST1 activation and caspase-3 and PARP cleavage, the EGFR inhibitor canertinib was ineffective at a similar concentration (Fig. 5b, c). We have previously shown that MST1 activates the mitochondrial pathway of cell death in β-cells through regulating the BH3-only protein member BIM of Bcl-2 family proteins and that MST1-induced apoptosis requires BIM to trigger mitochondrial-mediated apoptosis in β-cells^11^. Correspondingly, our data show that the EGFR/MST1 inhibitor neratinib but not the EGFR inhibitor canertinib significantly reduced downstream mitochondrial BIM induction under diabetic conditions (Fig. 5b, c). Unlike neratinib, canertinib showed a significant stimulatory effect on apoptotic effectors cleaved caspase-3, cleaved PARP, and BIM under basal conditions (Fig. 5b, c). These data suggest that EGFR signaling inhibition (as represented by canertinib) is dispensable for neratinib-induced MST1 inhibition and protection from apoptosis.

### Neratinib restores glycemia in a T1D mouse model

Initial pharmacokinetic studies in mice (Supplementary Fig. 6a) were performed to determine the exposure and half-life of neratinib. Mice were food-deprived overnight, and neratinib (5 mg/kg in 30% PEG400/0.5% Tween80/5% propylene glycol in saline; single i.p. dose) was given to the mice, and plasma samples were collected 30 min., 1, 3, and 7 h post dosing. Neratinib displayed a stable exposure profile in mice plasma and reached the maximum concentration at 306 ng/mL, on average 0.67 h post dosing (Supplementary Fig. 6a).

**Fig. 6:**
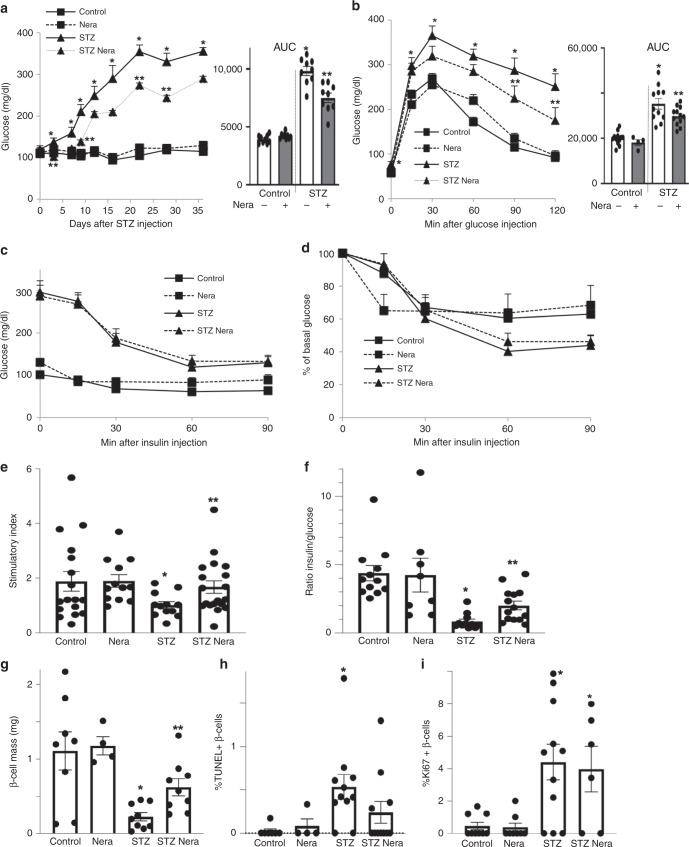
Neratinib improves glycemia, insulin secretion, and β-cell survival in the MLD–STZ-mouse model of type 1 diabetes. C57Bl/6J mice were injected with 40 mg/kg streptozotocin or citrate buffer for 5 consecutive days. Neratinib or vehicle was daily i.p. injected at a concentration of 5 mg/kg starting 3 h before the first STZ injection throughout the whole experiment of 35 days. **a** Random fed blood glucose measurements after the first STZ injection (day 0) over 35 days and **b** intraperitoneal glucose tolerance test (ipGTT); (respective area-under-the curve (AUC) analyses are shown in the right insets). **c**, **d** Intraperitoneal insulin tolerance test (ipITT). In **d**, basal glucose values were normalized to 100%. **e** Insulin secretion measured from retro-orbital blood draw during an ipGTT measured before (0 min) and 15 min after glucose injection; data are expressed as the ratio of secreted insulin at 15 min/0 min (stimulatory index). **f** The ratio of secreted insulin and glucose is calculated at fed state. **g**–**i** Mice were killed at day 35. **g** β-cell mass (given as percentage of the whole pancreatic section from ten sections spanning the width of the pancreas) and quantitative analyses from triple staining for **h** TUNEL or **i** Ki67, insulin, and DAPI expressed as percentage of TUNEL- or Ki67-positive β-cells ±SEM. An average number of 2331 (**h**), or 26369 (**i**) β-cells were counted. *n* = 9 mice/group for (**a**); *n* = 4–12 for (**b**); *n* = 5–15 for (**c**) and (**d**); *n* = 12–19 for (**e**); *n* = 8–14 for (**f**); *n* = 4–9 for (**g**); *n* = 4–11 for (**h**); and *n *= 6–11 mice/groupfor (**i**); all from three independent experiments). Data show means ± SEM. *p < 0.05 STZ compared with vehicle-injected mice, **p < 0.05 STZ compared with STZ-Nera injected mice; by one-way ANOVA with Bonferroni corrections for (**a**, **b**); by Student’s *t* tests for (**e**–**i**). Source data are provided as a Source Data file

Multiple low-dose streptozotocin (MLD–STZ) injections induce severe diabetes through activation of cell-intrinsic apoptotic pathways as well as selective immune-mediated destruction of β-cells. Since neratinib blocked apoptosis in human islets and in β-cells, we tested its ability to restore normoglycemia in vivo in a mouse model of MLD–STZ-induced β-cell demise and T1D. Neratinib treatment had no influence on body weight (Supplementary Fig. 6b). By day 3 of post-STZ treatment, hyperglycemia was evident, with glucose levels progressively increasing throughout the 35-day study (Fig. 6a). This was accompanied with severely impaired glucose tolerance in the STZ-treated control mice (Fig. 6b). Neratinib-treated mice had lower glucose levels during the entire 35 days of the study and exhibited significantly improved glucose tolerance. Neratinib had no effect on glycemia in nondiabetic control mice (Fig. 6a, b).

Insulin tolerance tests demonstrated dramatically elevated glucose levels during a 4-h fast prior to insulin administration (Fig. 6c), but neither neratinib nor STZ had a significant effect on insulin sensitivity, shown by the analysis of normalized glucose levels (Fig. 6d). Indeed, impaired insulin secretion observed in STZ-treated mice during an intraperitoneal glucose-stimulated insulin secretion assay was significantly improved with neratinib treatment (Fig. 6e). Consistently, insulin-to-glucose ratios were significantly elevated in neratinib-treated STZ mice (Fig. 6f). These data suggest that glucoregulatory effects of neratinib are primarily mediated by improved β-cell function. Furthermore, islet architecture was disrupted, leading to significantly reduced β-cell mass, in STZ-treated mice compared with nondiabetic control mice (Fig. 6g), as a result of profound increase in β-cell apoptosis (Fig. 6h and Supplementary Fig. 7). Together with the increased β-cell apoptosis induced by STZ, β-cell proliferation was also induced, indicative of compensatory capacity in response to STZ-induced β-cell injury (Fig. 6i and as reported before^11^). Neratinib restored β-cell mass and reduced β-cell apoptosis (Fig. 6g, h), with no effect on β-cell proliferation in either control or diabetic mice (Fig. 6i).

### Neratinib restores PDX1, NKX6.1, and Glut2 expression

**Fig. 7:**
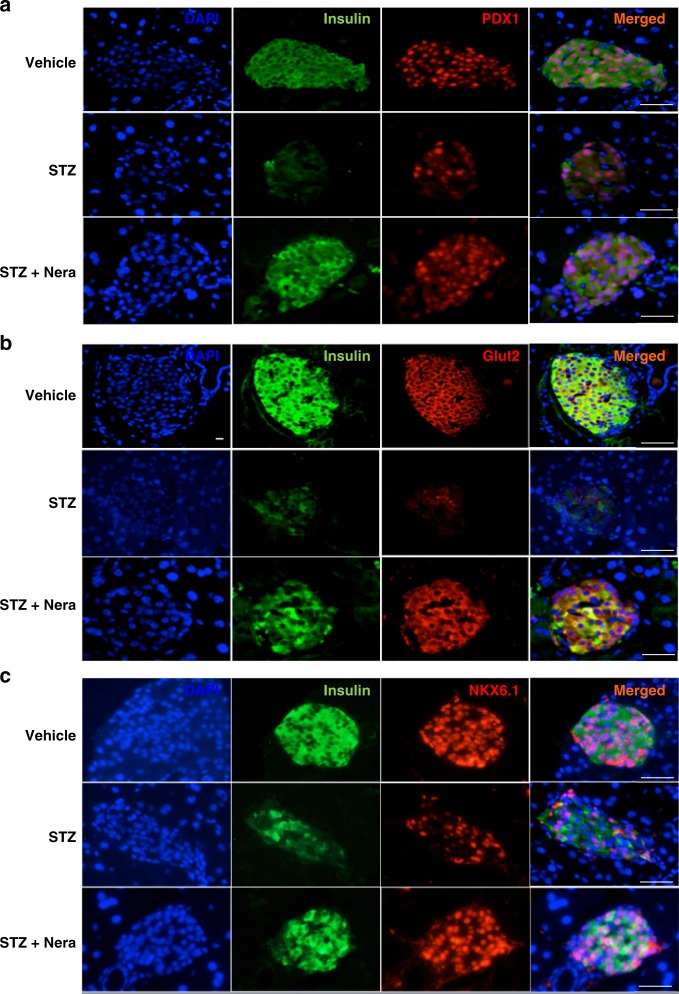
Neratinib restores PDX1, NKX6.1, and Glut2 expression. Representative triple stainings for PDX1 (red, **a**), Glut2 (red, **b**), or NKX6.1 (red, **c**), insulin (green), and DAPI (blue) are shown from vehicle-, STZ-, and STZ/Nera-treated mice (*n* = 8, 4, 9 & 9 mice/group). Scale bar, 100 μm

We next examined whether neratinib can also restore expression of three important markers for β-cell glucose metabolism and insulin production—the transcription factors PDX1 and NKX6.1 and the glucose transporter Glut2. MLD–STZ-treated mice showed impairment in the islet architecture with smaller islets and many insulin-negative cells together with reduced numbers and expression of nuclear PDX1- and NKX6.1-positive cells. Many cells within the islets, which still express insulin, had lost their NKX6.1 expression. These effects were markedly attenuated by neratinib treatment (Fig. 7a, b). The PDX1 target gene Glut2 was largely preserved in β-cell membranes of control mice, while the disrupted islet architecture of diabetic mice was also apparent by Glut2 staining, which was barely detectable in the MLD–STZ-treated mice (Fig. 7c). Neratinib treatment restored β-cell Glut2 expression (Fig. 7c). These effects of neratinib confirm previous results from MST1-KO mice, where PDX1 and Glut2 protein expression was greatly restored, and β-cell function and survival were highly preserved^11^.

### Neratinib restores glycemia in a T2D mouse model

**Fig. 8:**
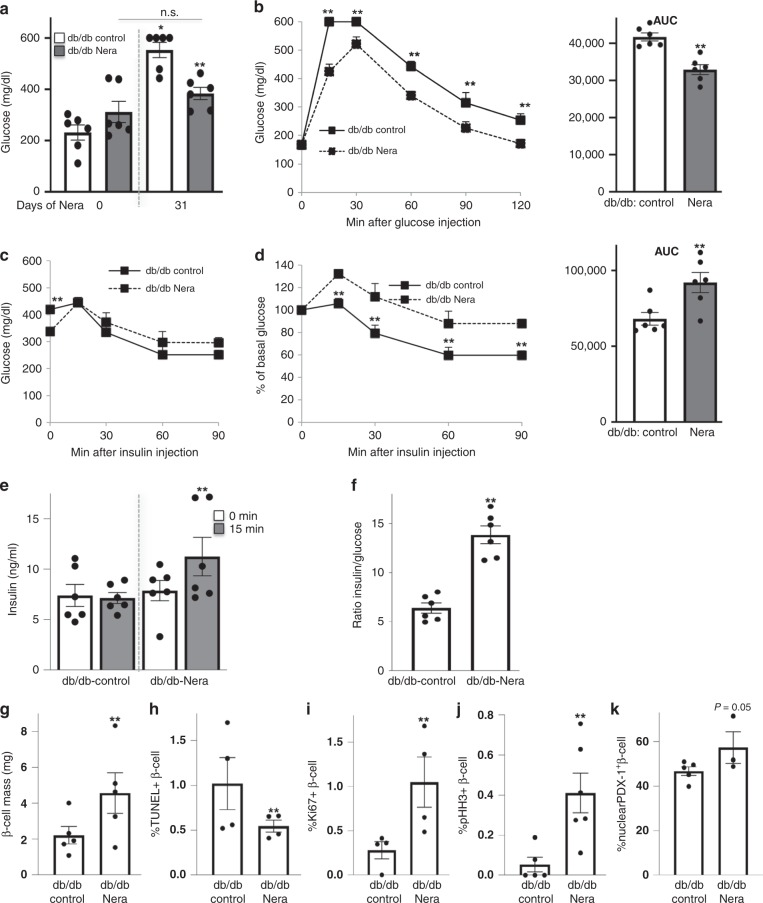
Neratinib improves glycemia, insulin secretion, and β-cell survival in obese db/db mouse model of type 2 diabetes. Obese diabetic Lepr^db/db^ mice on the C57BLKS/J background (db/db) were randomized in two groups at the age of 6 weeks, and then, neratinib or vehicle was daily i.p. injected at a concentration of 5 mg/kg throughout the whole experiment of 31 days. **a** Random fed blood glucose measurements before and after 31 days of Neratinib or vehicle injection (last day of the study). **b** Intraperitoneal glucose tolerance test (ipGTT). **c**, **d** Intraperitoneal insulin tolerance test (ipITT). In **d**, basal glucose values were normalized to 100% (respective area-under-the-curve (AUC) analyses for b and d are shown in the right insets). **e** Insulin secretion during an ipGTT measured before (0 min) and 15 min after glucose injection. **f** The ratio of secreted insulin and glucose is calculated at fed state. Data are representative of six mice per group (*n* = 6 for (**a**–**f**)). **g**–**j** Mice were killed at day 31. **g** β-cell mass (given as percentage of the whole pancreatic section from ten sections spanning the width of the pancreas; *n* = 5 mice/group) and quantitative analyses from triple stainings for (**h**) TUNEL, (**i**) Ki67, (**j**) pHH3, and (**k**) nuclear PDX1 expression, insulin, and DAPI expressed as percentage of TUNEL-, Ki67-, pHH3-, or nuclear PDX1-positive β-cells ± SEM. An average number of 13667 (**h**), 5937 (**i**), 7108 (**j**), or 4330 (**k**) β-cells were counted from *n* = 3–5 (**h**, **i**, **k**) or *n* = 5–6 (**j**) mice/group. Data show means  ± SEM. *p < 0.05 vehicle control at the end (10.5 weeks of age) compared with the start of the study (6 weeks of age), **p < 0.05 db/db compared with db/db-Nera injected mice; by one-way ANOVA with Bonferroni corrections for (**a**); by Student’s *t* tests for (**b**–**k**). Source data are provided as a Source Data file

Obese diabetic Lepr^db/db^ mice (db/db, Fig. 8) become severely diabetic and exhibit β-cell apoptosis and dysfunction^40^ together with islet upregulation of activated MST1^11^ at the age of 10 weeks. To test whether MST1 inhibition by neratinib could affect glycemia in the db/db model, 6-week-old obese db/db mice were treated daily with neratinib or vehicle over a period of 31 days. Neratinib treatment had no influence on body weight (Supplementary Fig. 6c). While blood glucose levels remained stable and glucose levels did not significantly increase in the neratinib-treated db/db mice after 4 weeks, they predictably rose to severe hyperglycemia (>500 mg/dL) in the vehicle-treated db/db mice (Fig. 8a). Attenuation of hyperglycemia was also evident in the intraperitoneal glucose tolerance test, in which neratinib showed lower glucose levels at all time points measured (Fig. 8b), and enhanced insulin secretion and insulin-to-glucose ratios (Fig. 8e, f). Basal glucose levels were unchanged by neratinib treatment after an overnight fast (Fig. 8b), but 20% lower in the neratinib-treated mice after a short fast of 4 h (Fig. 8c). These differences in fasting glucose prompted us to conduct an intraperitoneal insulin tolerance test, which showed that neratinib-treated mice had a modestly reduced ability to lower their glucose levels in response to insulin challenge (Fig. 8c, d), despite neratinib’s improvement in the overall restoration of glucose homeostasis. At the level of the β-cell, neratinib showed increased β-cell mass (Fig. 8g), which resulted from significantly reduced β-cell apoptosis (Fig. 8h and Supplementary Fig. 8a) and increased proliferation as determined by two markers Ki67 and phospho-Histone H3 (pHH3) immunolabeling (Fig. 8i, j and Supplementary Fig. 8b, c). While less than 50% of β-cells contained PDX1 in the nucleus, nuclear PDX1 was enhanced by neratinib (Fig. 8k and Supplementary Fig. 9).

### Neratinib improves β-cell survival in an ex vivo approach

**Fig. 9:**
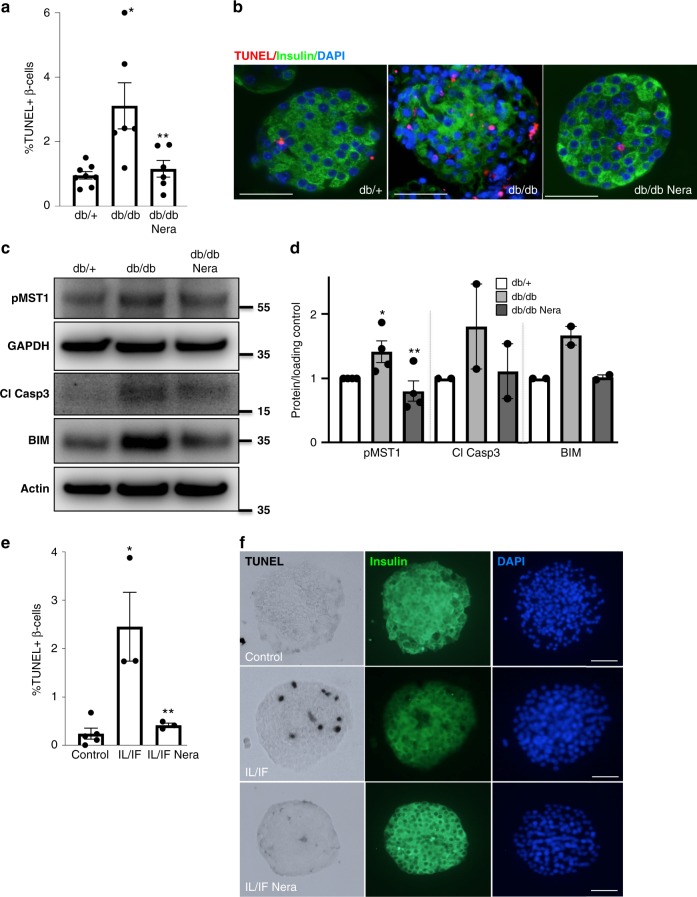
Neratinib improves β-cell survival in islets in a therapeutic ex vivo approach. **a**, **b** Isolated islets from 10-week-old obese diabetic Lepr^db/db^ mice or their heterozygous db/+ littermates were exposed to 10 μM neratinib for 24 h, fixed, and 2-μm sections were prepared. **a** Percentage of TUNEL-positive β-cells is shown as means ± SEM. **b** β-cell apoptosis was analyzed from islet sections by triple staining of TUNEL (red nuclei), insulin (green), and dapi (blue); scale bar, 100 μm. **c** Phospho-MST1 (pMST1; pThr183), caspase-3 cleavage, BIM and GAPDH, or actin were analyzed by western blotting shown by a representative blot (**c**) and pooled quantitative densitometry analysis (**d**). **e**, **f** Isolated islets from 2-month-old WT C57Bl/6 mice were exposed to the IL-1β/IFNγ cytokine mixture for 72 h (IL/IF); 10 μM neratinib was added to the culture for the last 24 h. **e** Percentage of TUNEL-positive β-cells is shown as means ± SEM. **f** β-cell apoptosis was analyzed from attached islet cultures by triple staining of TUNEL (black nuclei), insulin (green), and dapi (blue). Scale bar, 100 μm. All data are means ± SEM from multiple mice/condition (*n* = 6–8 for (**a**); *n* = 2–4 for (**c**); *n* = 3–5 for (**e**)). *p < 0.05 db/db or IL/IF to heterozygous db/+ or untreated control islets, **p < 0.05 neratinib to vehicle-treated islets under the same diabetogenic conditions; all by Student’s *t* tests. Source data are provided as a Source Data file

While neratinib improved glycemia in db/db mice in vivo, we aimed to confirm whether this effect occurs directly in islets ex vivo. Thus, we isolated mouse islets from severely diabetic db/db mice, where β-cell apoptosis was 3.2-fold increased, compared with islets from nondiabetic db/+ littermates. Adding neratinib to the islet cultures fully restored β-cell survival (Fig. 9a, b). MST1 activation and proapoptotic BIM in islets were reversed by ex vivo neratinib treatment (Fig. 9c, d). Neratinib also enhanced nuclear PDX1 expression (Supplementary Fig. 10).

We then addressed the question whether cytokine-induced β-cell apoptosis can also be normalized by neratinib and designed another therapeutic approach by treating the islets with the cytokine mixture of IL-1β/IFNγ cytokine mixture for 48 h and then added neratinib to the culture for another 24 h. While the cytokines induced a dramatic increase in β-cell apoptosis, neratinib fully restored β-cell survival (Fig. 9e, f).

### Neratinib is enriched and distributed in the pancreas

To provide further evidence that neratinib mediates the antidiabetic effects through a direct impact on the β-cell, we sought to confirm meaningful pharmacokinetic exposure of neratinib in the pancreas by using marix-assisted laser desorption ionization imaging mass spectrometry (MALDI-IMS)^41^. Specificity of the signal was tested on a liver phantom model, where neratinib was spotted on frozen mouse liver sections at concentrations ranging from 0 to 500 pmol/µl and peaks analyzed by MALDI imaging MS (Supplementary Fig. 11a). Starting at a concentration of 50 pmol/µl, neratinib could clearly be detected on liver sections at m/z 557.2, which represents the monoisotopic peak of neratinib (Supplementary Fig. 11a). Linear regression of signal intensity to neratinib concentration could be observed until 400 pmol/µl. The mass spectrum of the neratinib standard (Supplementary Fig. 11c) showed the expected isotope distribution as the simulated spectrum for neratinib (Supplementary Fig. 11b). Note the high abundance of the third peak at m/z 559.2, which is mostly caused by the stable isotope ^37^Cl, which occurs in nature with an abundance of 24.2% (sum formula of neratinib C_30_H_29_ClN_6_O_3_). This characteristic isotope distribution could also be detected in single spectra of high neratinib-intense regions in the pancreas after treatment (Supplementary Fig. 11d). A specific signal was obtained in pancreas tissue sections after a 4-week daily i.p. injection in the db/db mice with no signal evident from vehicle-treated control mice (Supplementary Fig. 11e). Neratinib was also clearly detected in pancreatic tissue 4 h after intraperitoneal (i.p.) injection of WT mice (Supplementary Fig. 11f).

## Discussion

In this study, we demonstrate neratinib as the inhibitor of MST1, a previously unappreciated activity alongside the dual inhibition of HER2/EGFR that drives its clinical utility in breast cancer. We show that neratinib protects β-cells from the apoptosis-inducing effects of a complex diabetic milieu in vitro in rat INS-1E β-cells and primary human and mouse islets, and lowers hyperglycemia in vivo in two widely used rodent models of diabetes. Repurposing of FDA-approved drugs has been a topic of great interest amidst the escalating costs of new drug development, particularly in the case of diseases with high-unmet medical need, such as T1D. Our studies suggest that neratinib—shown to be safe and well-tolerated in thousands of subjects in many Phase II and III clinical trials for cancer therapy^32,42^—could have a therapeutic effect in treating diabetes. Although diarrhea, vomiting, and nausea were most common neratinib-associated adverse events, they showed no increased risk of long-term toxicity or adverse consequences^32^. The appropriateness of the tolerability profile of neratinib for patients with a non-immediate-life-threatening disease such as diabetes must be considered carefully; as such, side effects may hinder its direct use in treating patients with either T1D or T2D, and thus, have to be reinvestigated in a clinical diabetes setting. Based on our mouse studies, a short therapeutical interval of 30 days could markedly restore β-cell survival and function, and thus, maybe sufficient for therapy in patients. Especially in the obese db/db model, it becomes clear that neratinib treatment prevented the severe increase in blood glucose over time. We started the experiment, when mice were already mildly hyperglycemic (mean random glucose of all mice was 271.5 mg/dl). After 30 days of therapy, the control group showed a 2.4-fold increase in blood glucose (from 231 to 554 mg/dl), while the neratinib group had no significant blood glucose increase (312–384 mg/dl).

Besides their potent action in cancer therapy, multitarget tyrosine inhibitors have been suggested for a long time for the treatment of diabetes, in some cases driven by polypharmacology outside of the tyrosine kinase target class. For example, imatinib, which targets c-Abl, DDR1/2, c-Kit, and PDGFR; sunitinib, which targets FLT1/3/4, c-Kit, PDGFR, and VEGFR2; erlotinib and PD153035, potent and specific inhibitors of EGFR, have all been reported to have potent anti-hyperglycemic effects in preclinical as well as in several clinical case reports^43,44^. In a related compound class, the tyrosine phosphatase PTP1b inhibitor ertiprotafib was explored as a novel insulin sensitizer for T2D, based on its ability to improve fasting blood glucose and glucose tolerance in the Zucker diabetic fatty rat^45^, with triglyceride and free fatty acid lowering effects mediated through inhibition of IκB kinase β^46^. With regard to EGFR inhibitors, two independent studies show profound reduction of fasting glucose levels and normalization of HbA1c in two lung cancer patients with T2D treated with the EGFR inhibitor erlotinib^47,48^. Although such cases were not reported with neratinib, one wonders whether the well-known polypharmacology of erlotinib may overlap with neratinib and thus exhibit antidiabetic effects.

EGFR signaling is associated with insulin resistance and liver, muscle, and adipose inflammation. EGFR inhibition through tyrosine kinase inhibition was suggested as therapy of insulin resistance, because its inhibition could restore insulin sensitivity by decreasing inflammation in insulin target tissue^43^. EGFR inhibition improved tyrosine phosphorylation levels of the insulin receptor and the insulin receptor substrate in obese mice, which are classically associated with improved insulin sensitivity; however, direct analysis of insulin sensitivity was not performed in these studies^44^.

Although FDA was approved for cancer therapy, further medicinal chemistry optimization to improve drug selectivity and remove its covalent linkage would be desirable to limit drug toxicity and to provide better specificity for a chronic indication like diabetes. Neratinib inhibits MST1 very potently; however, it targets many other kinases, as the development of kinase inhibitor has been challenging^38,49^. A foundation for kinase inhibitor biology and toxicity has been set by an excellent previous study, which also reported potent activity of neratinib (also formerly known as HKI-272) on MST family members^38^. Studies to improve MST1 specificity and potency are currently under way.

While neratinib is not at all selective for MST1, we believe that the underlying mechanisms of diabetes protection still derive from a direct protective effect on the β-cell mediated by MST1 and not from an effect on insulin sensitivity mediated by EGFR. Improved glucose tolerance and insulin secretion were observed in both models, while insulin sensitivity was unaffected by neratinib in the STZ model and modestly impaired in the obese insulin-resistant db/db model, which was only evident after normalization of the fasting glucose levels. Without such normalization applied, glucose levels during the insulin tolerance test were not significantly affected by neratinib, despite the significant glucose reduction in neratinib-treated mice after 4 h of fasting. Also, neratinib almost fully blocked β-cell apoptosis in human islets induced by MST1 overexpression and also had no additive effect in islets isolated from MST1-KO mice, in which apoptosis was already blocked by the genetic disruption of MST1 itself.

A general concern in targeting the reduction of β-cell apoptosis and the induction of β-cell proliferation in diabetes therapy is the potential for uncontrolled expansion of multiple cell types and oncogenic transformation. Organ-specific double knockout of MST1/2, e.g., in the liver has been shown to lead to tumor growth, because of the lack of constraints on cellular proliferation in these mice driven by the Hippo-YAP pathway^50^. Although neratinib shows some modest selectivity toward MST1 than to MST2, one of our long-term objectives will be to generate an MST1 inhibitor with greater selectivity versus MST2 than neratinib (e.g., more than 50-fold). In our previous work, we carefully explored the phenomenon of β-cell proliferation in both the global as well as β-cell-specific MST1-knockout mice. At basal normoglycemic level, there was no change in β-cell proliferation in the MST1-KO, but there was increased β-cell proliferation under conditions of STZ-induced hyperglycemia both in the global as well as β-cell-specific MST1-KO mice. Also, β-cell-specific MST1 ablation fosters β-cell compensatory hyperplasia in HFD-treated mice^11,24^. This suggests that there is no proliferative potential of MST1 inhibition under non-stressed conditions, but the capacity to overcome β-cell stress by increased proliferation and reduced β-cell apoptosis is triggered under circumstances of such stress, leading to reestablishment of glucose homeostasis. Similarly, neratinib did not affect apoptosis or proliferation in nondiabetic mice, but increased β-cell proliferation in db/db mice together with profound reduction in β-cell apoptosis, leading to β-cell mass compensation and improved glycemia. However, in the absence of CRISPR-mediated deletion of all other potential neratinib-sensitive kinases, similarly as done previously^51^ we cannot formally exclude off-target effects and definitively assign MST1 as the only target of neratinib in this study.

Overall, basal β-cell proliferation is very limited in mouse islets and negligible in human islets^52^. This is probably an evolved property that protects from insulin production during long times of starvation. This proliferative incapacity can be attributed at the molecular level to the loss of YAP, the major downstream component of the Hippo-YAP pathway, which controls organ development and size^37,53–55^. YAP disappears exactly at the point of islet development, when Ngn3 becomes present in order to drive endocrine cell differentiation^37,56,57^. Therefore, MST1 inhibition in the absence of YAP expression would not lead to tumor development. The Hippo-YAP pathway acts in coordination with many other cell-size and proliferation-determining factors in development, including insulin-like growth factor (IGF1R^58^) and EGF receptor signaling (EGFR^59^). Such interaction can even be cell-autonomous, as shown in Drosophila, where YAP-EGFR crosstalk promotes proliferation in neighboring cells^60^. Thus, we cannot rule out the possibility that simultaneous EGFR inhibition could be important for limiting the oncogenic potential of MST1 and MST2 inhibition and subsequent activation of YAP directed transcription.

Several MST1 inhibitors have been identified recently^61^. Compound 9E1, the first small-molecule MST1 inhibitor identified from an organometallic library screen^27^, showed strong off-target effects on other kinases such as proto-oncogene serine/threonine protein kinase PIM1 (PIM-1) and glycogen synthase kinase 3 (GSK-3β)^27^, and thus did not enter any preclinical studies. MST1 inhibition by LP-945706 has anti-inflammatory efficacy in an experimental autoimmune encephalomyelitis (EAE) model^29^. The reversible and selective MST1/2 inhibitor XMU-MP-1 promotes tissue repair and regeneration by cellular proliferation induction in human liver cells and in a mouse model of liver and intestine injury^28^. It will be interesting to test the efficiency of the novel MST1 inhibitors LP-945706 or XMU-MP-1 to promote β-cell survival exposed to a complex diabetic milieu or in diabetic mice in comparison with neratinib.

This study shows the beneficial effects of the kinase inhibitor neratinib in ameliorating hyperglycemia as well as improving β-cell survival and function under diabetogenic conditions. The subject of our ongoing work in this regard is the design of neratinib-based MST1 inhibitors that exhibit enhanced potency and selectivity for MST1, with safety profiles commensurate with the chronic treatment of diabetes. The identification of neratinib as an MST1 inhibitor thus amounts to an accelerated path to a preclinical proof of concept, shown herein, as well as a firm basis for a follow-on medicinal chemistry optimization program aimed at retaining the drug-like properties of neratinib but improving upon its selectivity and safety.

## Methods

### Cell culture, treatment, and islet isolation

Human islets were isolated from eight pancreases of nondiabetic organ donors at PRODO Labs and at Lille University and cultured on extracellular matrix-coated dishes (Novamed, Jerusalem, Israel)^62^ or on Biocoat Collagen I coated dishes (#356400, Corning, ME, USA). Islet purity was greater than 95% as judged by dithizone staining (if this degree of purity was not achieved by routine isolation, islets were handpicked). Islets from MST1-knockout (MST1-KO) mice and their WT littermates^63^ were isolated by pancreas perfusion with a Liberase TM (#05401119001, Roche, Mannheim, Germany) solution^62^ according to the manufacturer’s instructions and digested at 37 °C, followed by washing and handpicking. The clonal rat β-cell line INS-1E was kindly provided by Dr. Claes Wollheim, Geneva & Lund University. Human islets were cultured in complete CMRL-1066 (Invitrogen) medium at 5.5 mM glucose, mouse islets and INS-1E cells at complete RPMI-1640 medium at 11.1 mM glucose^11^. Mouse macrophage Raw264.7 cell line was purchased from ATCC and cultured in DMEM (Gibco) supplemented with 10% FBS. Islets and INS-1E were exposed to complex diabetogenic conditions: 22.2–33.3 mM glucose, 0.5 mM palmitic acid, the mixture of 2 ng/ml recombinant human IL-1β (R&D Systems, Minneapolis, MN) + 1000 U/ml recombinant human IFN-γ (PeProTech) for 24–72 h, 100 μM H_2_O_2_ for 6 h, or 0.1, 1 mM thapsigargin for 6 or 24 h. Neratinib or vehicle (0.1% DMSO) was added to the cell culture 1–2 h before treatment. Palmitic acid (Sigma) was dissolved at 10 mmol/l in RPMI-1640 medium containing 11% fatty acid–free bovine serum albumin (BSA) (Sigma) under an N2-atmosphere and added to the culture at 0.5 mM^64^. In some experiments, human or mouse islets or INS-1E cells were additionally cultured with various concentrations of neratinib (Calibr). Isolated human islets or INS-1E cells were infected with adenovirus carrying LacZ as control or MST1 or LATS2 (all Vector Biolabs), at a multiplicity of infection of 100 (human islets) or 20 (INS-1E) for 4 h^11^. Adenovirus was subsequently washed off with PBS and replaced by fresh medium with 10% FBS and the respective analysis performed after 48 h post infection.

All human islet experiments were performed in the islet biology laboratory, University of Bremen. Ethical approval for the use of human islets had been granted by the Ethics Committee of the University of Bremen. The study complied with all relevant ethical regulations for work with human cells for research purposes. Organ donors are not identifiable and anonymous; such approved experiments by using human islet cells for research are covered by the NIH Exemption 4 (Regulation PHS 398). Human islets were distributed by the two JDRF and NIH-supported approved coordination programs in Europe (Islet for Basic Research program; European Consortium for Islet Transplantation ECIT) and in the United States (Integrated Islet Distribution Program IIDP)^65^.

### High-throughput screening and hits confirmation

High-throughput screening targeting MST1 was conducted in low-binding 1536 microplate (Corning, NY) by using LanthaScreen Eu Kinase Binding assay. In total, 641 annotated compounds in a Kinase inhibitor library (Calibr) were screened at 1, 0.2, 0.04, 0.008, and 0.0016 µM, with staurosporine as the positive control. Compounds in 1000× DMSO stock solution were dispensed by using Echo555 liquid dispensing system (Labcyte, CA) to 1536-well microplate (Corning, NY). Kinase buffer A (ThermoFisher, MA) was prepared and added to each well. MST1 kinase (ThermoFisher) and Eu-anti-GST Antibody (ThermoFisher) was prepared at 15 nM and 6 nM, respectively. Kinase tracer 222 (ThermoFisher) was prepared at 300 nM. Each reagent was added to the microplate in equal volume. Plates were incubated at room temperature for 1.5 h in the dark, scanned on Envision plate reader with excitation at 340 nM, emission at 665 and 615 nM. Data were analyzed based on the emission ratio of 665 nm/615 nm, normalized to DMSO as negative control. The criteria of picking primary hits was ≥75% inhibition at 1 µM and ≥50% inhibition at 0.2 µM, compared with staurosporine (1 µM), which is considered 100% inhibition at 1 µM. For hit confirmation, primary hits were assayed in triplicates with 12 points in a dose-dependent manner, starting at a neratinib dose of 5 µM followed by 1:3 serial dilution.

### Nanosyn kinase profiling

Neratinib was tested at Nanosyn (Santa Clara, CA) in a panel of 50 biochemical kinase assays identified in Fig. 1c at 10 μM, and later on, in a panel of 250 biochemical kinase assays (Supplementary Fig. 1a) at 3 μM in duplicate wells. A selected set of kinases where more than 90% of inhibition was observed at 3 μM was retested in dose response for neratinib and IC_50_ was determined (Supplementary Fig. 1b). The testing was performed by using microfluidics mobility shift assay technology using ATP concentrations at Km level of each kinase. Data presented as average from the two duplicate wells.

### Cytotoxicity CellTiter-Glo® assay

INS-1E cells were treated with compounds in a dose-dependent manner in 384-well microplates (Corning, NY) at 10^4^ cells/well in 25 µL of complete grow medium. After 24 h of compound treatment, 5 µL of Celltiter-Glo^®^ reagent (Promega, WI) was added to each well. Assay plates were shaken vigorously for 1 min at RT to achieve completed cell lysis. Luminescence intensity was detected on Envision plate reader (Perkin Elmer, MA).

### Caspase-3 activation Nucview assay

INS-1E cells were treated with compounds in a dose-dependent manner in 384-well microplates (Corning, NY) at 10^4^ cells/well. Apoptosis was induced after 24 h of compound treatment by 0.1 µM Thapsigargin (Torics, Bristol, United Kingdom) with caspase-3 substrate, Nucview 488 (Biotium, CA) in the treatment. Sixteen hours later, cells were fixed in 3% paraformaldehyde (Electron Microscopy Sciences, PA) and stained with Hoechst33342 (ThermoFisher). Data analysis was based on the fluorescence intensity of Nucview488 and Hoechst33342. Similarly, in caspase-3 activation assay induced by the cytokine mixtures in high-glucose conditions, INS-1E cells were exposed up to 6.7 µM of neratinib for 2 h followed by 16 h of induction in 100 ng/mL of TNFα and 200 ng/mL of IFNγ with 33 mM glucose in assay medium. Caspase-3 activity was evaluated through Nucview488 and Hoechst33342 staining.

### RT-PCR assay

Mouse macrophage Raw264.7 cells were treated with vehicle (0.1% DMSO) or neratinib in triplicates at different concentrations for 2 h, followed by 100 ng/mL LPS stimulation for 4 h. Cells were washed and RNA isolated by using RNeasy Mini Kit (Qiagen). One microgram of cDNA of each treatment sample was synthesized by using SuperScript III First-Strand Synthesis (Invitrogen). Taqman mouse primers were purchased from ThermoFisher: TNFα (Catalog No. Mm00443258_m1), IL-6 (Catalog No. Mm00446190_m1), IL-1β (Catalog No. Mm00434228_m1), and GAPDH as endogenous housekeeping control (Catalog No. Mm99999915_g1). The qPCR reaction was set up for TNFα, IL-6, IL-1β, and GAPDH individually, with technical triplicates for each gene per treatment sample and performed by the Applied Biosystems ViiA 7 real-time PCR system. The ∆∆CT method was used to analyze the relative changes in gene expression.

### CETSA assay

For the CETSA assay^39^, INS-1E cells were treated with 5 μM neratinib or canertinib for 2 h in the CO_2_ incubator at 37 °C in a 6-well plate. Thereafter, cells were pelleted at 200 g for 4 min and resuspended in PBS supplemented with phosphatase and protease inhibitor cocktail at the cell density of 3 Mill./100 µl. Each cell suspension was distributed into five 0.2-ml PCR tubes with 100 µl of cell suspension per tube. PCR tubes were heated at their designated temperature (43–55 °C) on a thermal cycler for 3 min, incubated at room temperature for 3 min, and snap-frozen in liquid nitrogen. Cell lysates were prepared by freezing–thawing the samples in liquid nitrogen twice, and soluble MST1 was detected by western blot analysis.

### Animals

For the MLD–STZ experiment, 8–10-week-old male C57BL/6J mice were i.p. injected with streptozotocin (STZ; 40 mg/kg; Sigma) freshly dissolved in 50 mM sodium citrate buffer (pH 4.5) or citrate buffer as control for 5 consecutive days (referred to as multiple low doses/MLD–STZ). Obese diabetic Lepr^db/db^ mice on the C57BLKS/J background (db/db) were obtained from Charles River at the age of 5 weeks and randomized in 2 groups at the age of 6 weeks. Neratinib or vehicle (30% PEG400/0.5% Tween80/5% propylene glycol in NaCl) was daily i.p. injected at a concentration of 5 mg/kg starting 3 h before the first STZ injection or at 6 weeks of age (db/db) throughout the whole experiment. Random blood was obtained from the tail vein of non-fasted mice, and glucose was measured by using a Glucometer (Freestyle; TheraSense Inc., Alameda, CA). Mice were killed at the end of the experiment, and their pancreases were isolated. Throughout the whole study, body weight was measured weekly. All animals were housed in a temperature-controlled room with a 12-h light/dark cycle and were allowed free access to food and water in agreement with NIH animal care guidelines, §8 German animal protection law, German animal welfare legislation, and with the guidelines of the Society of Laboratory Animals and the Federation of Laboratory Animal Science Associations. All protocols were approved by the Bremen Senate (Senator for Science, Health, and consumer protection), and we have complied with all relevant ethical regulations for animal testing and research.

### Glucose and insulin tolerance tests, insulin secretion

For intraperitoneal glucose tolerance tests, mice were fasted 12 h overnight and injected i.p. with glucose (40%; B.Braun, Melsungen, Germany) at a dose of 1 g/kg body weight. Blood samples were obtained at time points 0, 15, 30, 60, 90, and 120 min for glucose measurements by using a Glucometer. For i.p. insulin tolerance tests, mice were injected with 0.75 U/kg body weight recombinant human insulin (Novolin, Novo Nordisk) after 4–5-h fasting, and glucose concentration was determined with the Glucometer. Insulin secretion was measured before (0 min) and after (15 and 30 min) i.p. injection of glucose (2 g/kg) and measured by using ultrasensitive mouse Elisa kit (ALPCO Diagnostics, Salem, NH).

### Immunohistochemistry

Mouse pancreases were dissected and fixed in 4% formaldehyde at 4 °C for 12 h before embedding in paraffin^62^. Two-four-micrometer sections were deparaffinized, rehydrated, and incubated overnight at 4 °C with anti-PDX-1 (Abcam; #47267), anti-Glut2 (Chemicon; #07-1402), anti-Ki67 (Dako; #M7249), anti-phospho-Histone H3 (Ser10; Merck #06-570), and anti-NKX6.1 (DSHB, University of Iowa #F55A12^66^) in combination with TSA (Invitrogen #T30955), or for 2 h at room temperature with anti-insulin (Dako; A0546) antibodies (all at a dilution of 1:100, except anti-PDX-1, which was diluted 1:400) followed by fluorescein isothiocyanate (FITC)- or Cy3-conjugated secondary antibodies (Jackson ImmunoResearch Laboratories, West Grove, PA). Slides were mounted with Vectashield with 4′6-diamidino-2-phenylindole (DAPI) (Vector Labs). β-cell apoptosis was analyzed by the terminal deoxynucleotidyl transferase-mediated dUTP nick-end labeling (TUNEL) technique according to the manufacturer’s instructions (In Situ Cell Death Detection Kit, TMR red; Roche) and double stained for insulin. Fluorescence was analyzed by using a Nikon MEA53200 (Nikon GmbH, Dusseldorf, Germany) microscope, and images were acquired by using NIS-Elements software (Nikon).

### Morphometric analysis

For morphometric data, ten sections (spanning the width of the pancreas) per mouse were analyzed. Pancreatic tissue area and insulin-positive area were determined by computer-assisted measurements by using a Nikon MEA53200 (Nikon GmbH, Dusseldorf, Germany) microscope, and images were acquired by using NIS-Elements software (Nikon). β-cell mass was obtained by multiplying the β-cell fraction by the weight of the pancreas.

### Western Blot analysis

At the end of the incubation periods, islets and INS-1E cells were washed in ice-cold PBS and lysed in RIPA lysis buffer containing 50 mM Tris HCl, pH 8, 150 mM NaCl, 1% NP-40, 0.5% sodium deoxycholate, and 0.1% SDS supplemented with Protease- and phosphatase inhibitors (Pierce, Rockford, IL, USA). Protein concentrations were determined with the BCA protein assay (Pierce). Equivalent amounts of protein from each treatment group were run on a NuPAGE 4–12% Bis-Tris gel (Invitrogen) and electrically transferred onto PVDF membranes. After blocking by 2.5% milk (Cell Signaling) and 2.5% BSA, membranes were incubated overnight at 4 °C with rabbit anti-cleaved caspase-3 (#9664), rabbit anti-PARP (#9532), rabbit anti-cleaved PARP (rat specific #9545), rabbit anti-phospho YAP(S127) (#4911), rabbit anti-LATS2 (#5888), rabbit anti-tubulin (#2146), rabbit anti-GAPDH (#2118), rabbit anti-β-actin (#4967) (all Cell Signaling Technology), and rabbit anti-PDX1 (#47267) and rabbit anti-p-MST1 (#79199) (both from Abcam) antibodies, all at a dilution of 1:1000, followed by horseradish-peroxidase-linked anti-rabbit IgG (Jackson). Membrane was developed by using a chemiluminescence assay system (Pierce) and analyzed using DocIT^®^LS image acquisition 6.6a (UVP BioImaging Systems, Upland, CA, USA). Uncropped and unprocessed scans of all Western blots are available in the Source Data file.

### In vitro luciferase assay

INS1-E cells were transfected with LATS-BS firefly luciferase reporter constructs by using jetPRIME transfection reagent (PolyPlus, Illkirch, France). pCDNA3.1neo-NLucYAP15 and pCDNA3.1neo-14-3-3-CLuc was a gift from Xiaolong Yang (Addgene plasmid # 107610; http://n2t.net/addgene:107610; RRID:Addgene_107610)^35^. As internal transfection control, pRL-Renilla luciferase control reporter vector (Promega) was co-transfected into each sample. Twenty-four hours after transfection, cells were transduced with Ad-h-LacZ, or Ad-h-LATS2 and Ad-h-MST1 (Vector Biolabs) for another 48 h. Neratinib or canertinib (10 µM) was added for the last 24 h. Thereafter, Western blot analysis (see above) and luciferase assay was performed by using Dual-Luciferase Reporter Assay System (Promega)^11^ in a parallel set of experiments. Luciferase signal was calculated based on the ratio of luciferase activity of LATS-BS to control reporter vector.

### Marix-assisted laser desorption ionization

MALDI imaging mass spectrometry (MALDI imaging MS) was performed on pancreas, liver, colon, stomach, kidney, heart, and brain tissue sections from WT C57BL/6J and db/db mice in triplicates. Neratinib distribution in the pancreas was studied after neratinib treatment for five days with a dosage of 5 mg/kg neratinib in WT control mice or after the 31-day treatment period in db/db mice; animals were killed 4 h after the last treatment. For MALDI imaging MS, 10-µm cryo sections were cut with a cryo-microtome (CM1860, Leica Biosystems, Nussloch, Germany) and mounted on indium–tin-coated conductive glass slides (Bruker Daltonics, Bremen, Germany). The matrix (HCCA in 50% ACN, 0.5% TFA) was applied with the ImagePrep Device (Bruker Daltonics), and MALDI spectra were recorded by using a Bruker autofleX speed mass spectrometer in positive reflector mode with a mass range of 400–1400 m/z. A large-size laser diameter was used with a lateral resolution of 100 µm, and 500 laser shots per pixel were accumulated with the random walk option set to 100 shots per position. For data analyses, the unprocessed raw data were imported into the Software SCiLS Lab, version 2016b (SCiLS GmbH, Bremen, Germany). The dynamic range of the neratinib signal was analyzed by using drug standards (0–500 pmol/µl). Standards were spotted on mice liver cryo-sections and the spectral intensity was plotted.

### Statistical analysis

Samples in different experiments were evaluated in a randomized manner by six investigators (D.A., A.D., K.A., R.H., B.L. and SG) who were blinded to the treatment conditions (Fig. 3c, d; 4d–g; 6g–i; 7g–j; 8a, b, e, f). Data are presented as means ± SEM unless otherwise stated with the number of independent individual experiments (biological replicates) or analyzed mice presented in the figure legends. Mean differences were tested by Student’s *t* tests. ANOVA for multiple group comparisons with Bonferroni corrections was performed for data in Figs. 6a, b and 7a. *P* values < 0.05 were considered statistically significant.

### Reporting summary

Further information on research design is available in the Nature Research Reporting Summary linked to this article.

## Supplementary information

Supplementary Information

Reporting Summary

Source Data

## Data Availability

All data generated or analyzed during this study are included in this published article and its supplementary information files. Source data are provided as a Source Data file. All additional data are available from the corresponding authors upon reasonable request.
