# Supplementary material for: Neratinib protects pancreatic beta cells in diabetes
Source: Nat Commun. 2019 Nov 1;10:5015. doi: 10.1038/s41467-019-12880-5 (PMC6825211; doi:10.1038/s41467-019-12880-5)
Supplement: Supplementary file 1 — Supplementary Information [file 41467_2019_12880_MOESM1_ESM.pdf]

Supplementary Figures

Neratinib Protects Pancreatic Beta Cells in Diabetes

Ardestani et al.

Supplementary Figure 1

a

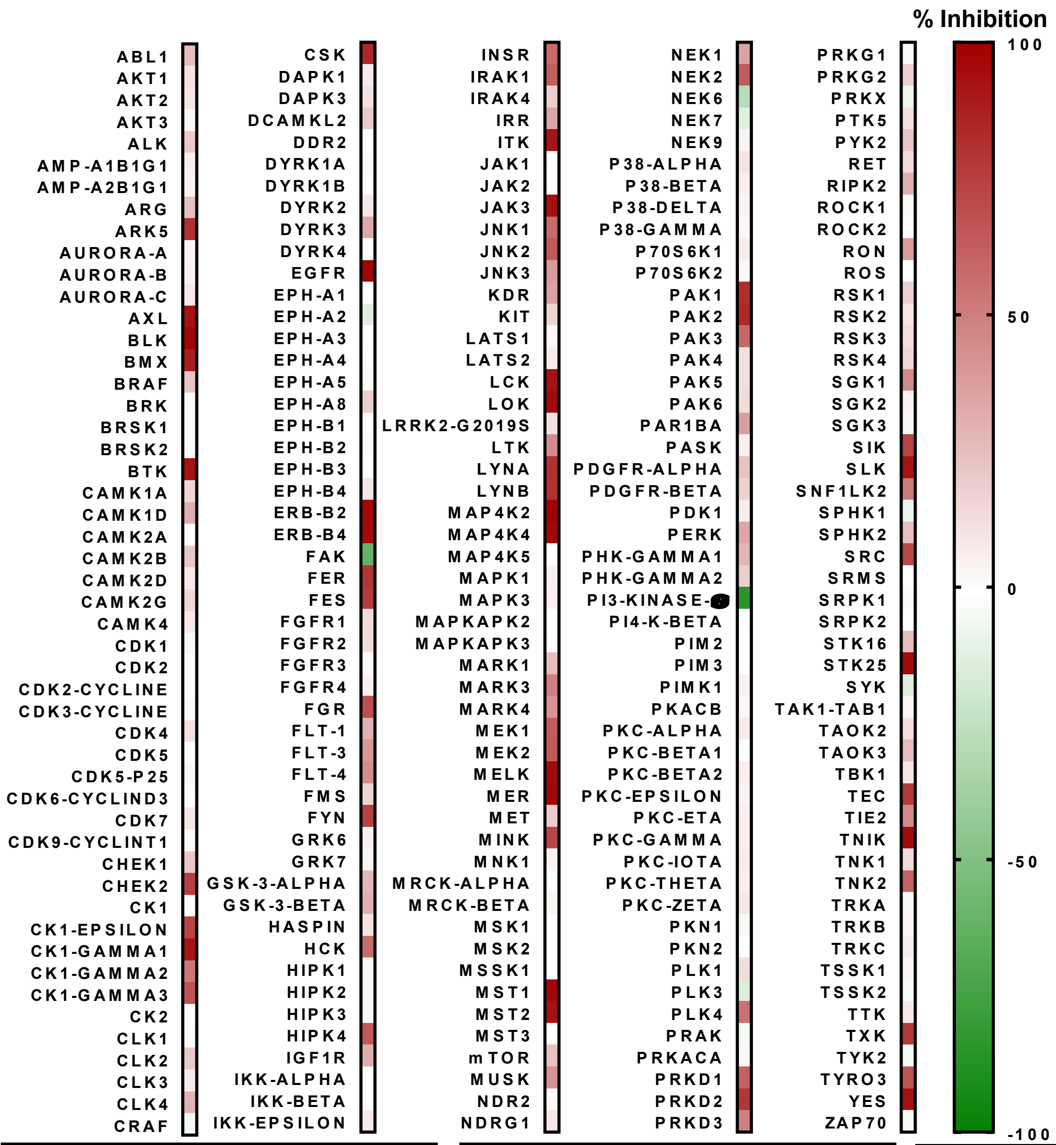

b

Kinases	Neratinib (IC_{50} uM)
IRAK1	2.48
LCK	0.066
LOK	0.066
LYNA	0.379
LYNB	0.304
MAP4K2	0.102
MAP4K4	0.062
MAP4K5	0.0001
MINK	0.943
MEK1	1.49
MEK2	1.2
MER	0.026
PAK1	0.609

Kinases	Neratinib (IC_{50} uM)
PAK2	0.408
PAK3	1.5
PAK4	>20
PAK5	>20
PAK6	>20
PHK1	5.85
MST1	0.091
MST2	0.21
MST3	0.005
SIK	1.05
SLK	0.177
SRC	0.727
STK25	0.05

Kinases	Neratinib (IC_{50} uM)
TNIK	0.081
YES	0.087
ARK5	0.583
AXL	0.185
CHEK2	0.937
EGFR	0.0003
FES	1.0
HCK	2.75
HIPK1	>20
HIPK4	1.66
MARK4	7.36
BLK	0.016

Supplementary Figure 1. Results of kinase profiling for Neratinib by Nanosyn. (A) Neratinib was assayed at 3 μM in duplicate wells against 250 biochemical kinase assays. % inhibition was determined for each assay. (B) Neratinib was assayed in a 12 points dose-dependent serial concentration and IC_{50} was determined against 38 biochemical kinases. Related to Figure 1.

Supplementary Figure 2

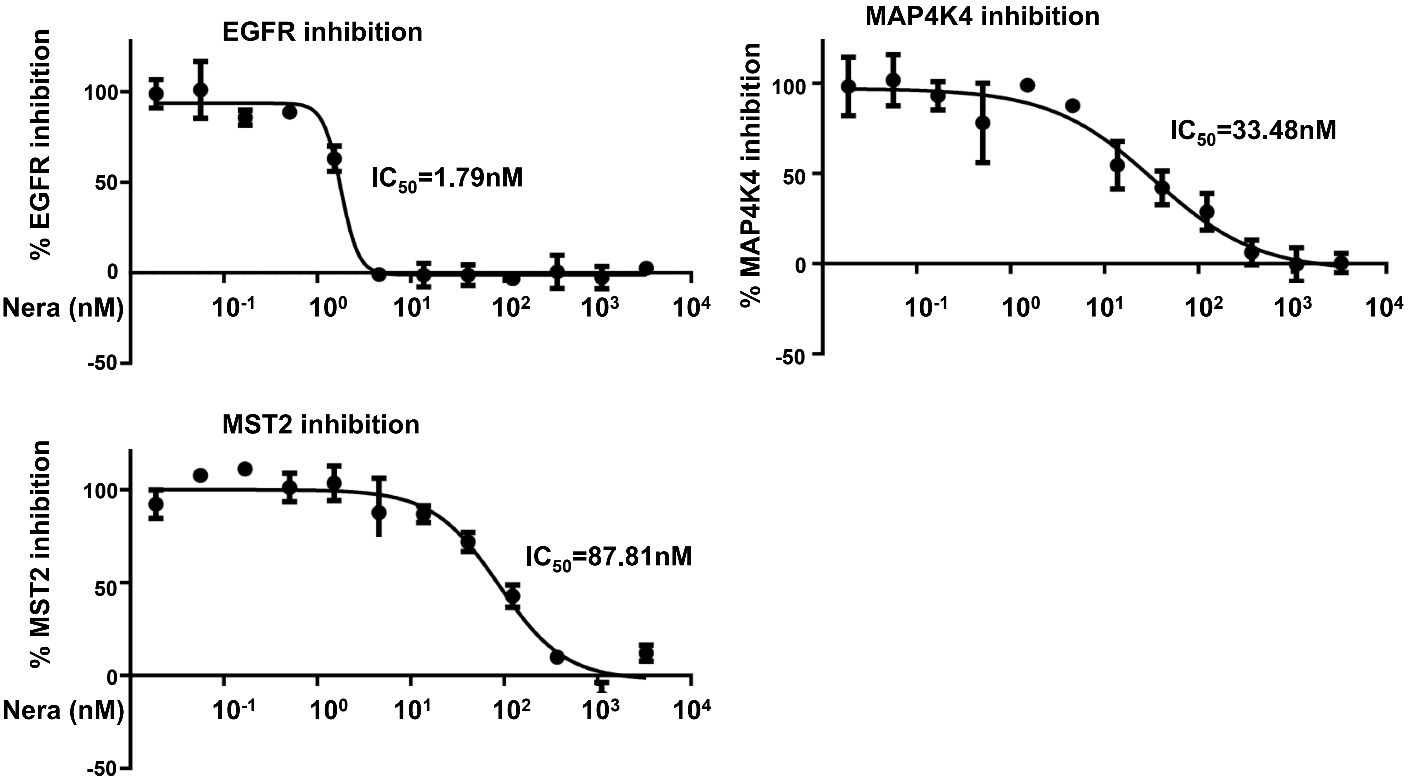

Supplementary Figure 2. Neratinib's effect on EGFR, MAP4K4 and MST2 inhibition. Biochemical dose response of EGFR, MAP4K4 and MST2 inhibition by neratinib. Data show means ±SEM from 3 independent experiments (n=3). Related to Figure 1.

Supplementary Figure 3

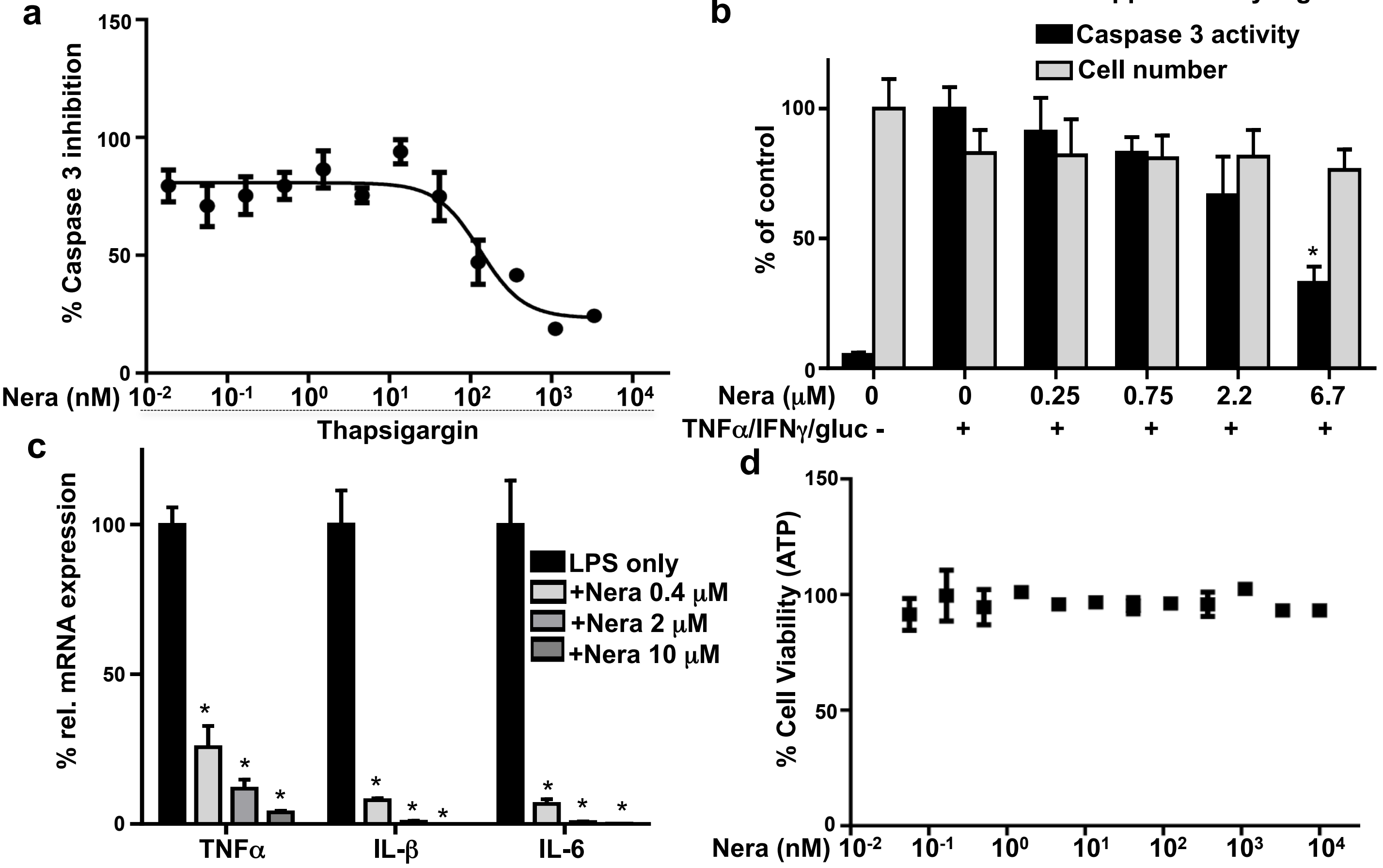

Supplementary Figure 3. Neratinib protects INS-1E β-cells from ER and cytokine induced stresses. (A) Caspase-3 activation induced by thapsigargin mediated ER stress. INS-1E cells were treated with compounds in a dose-dependent manner at 10^4 cells/well. Apoptosis was induced after 24 hours by 0.1 μM thapsigargin with caspase-3 substrate Nucview488. 16 hours later, cells were fixed in 3% paraformaldehyde and stained with Hoechst33342. Data analysis is based on the fluorescence intensity of Nucview 488 and Hoechst33342, with normalization to cell cytotoxicity, which was evaluated through Celltiter-Glo. (**B**) Caspase-3 activation induced by cytokine mixture at high glucose. INS-1E cells were exposed up to 6.7μM of neratinib for 2h followed by 16h of induction in 100 ng/mL of TNFα and 200 ng/mL of IFNγ with 33 mM glucose in assay medium. Caspase-3 activity was evaluated through Nucview488 and Hoechst33342. **(C)** Anti-inflammatory effect of neratinib through gene expression assay (RT-PCR). Mouse macrophage Raw264.7 cells were treated with neratinib in different concentrations for 2 hours, and followed by 100 ng/mL LPS stimulation for 4 hours. Cells were harvested and gene expression of TNFα, IL-6 and IL-1β were analyzed through qRT-PCR. **(D)** Cytotoxicity of neratinib evaluated by CellTiter-Glo®. INS1E cells were treated with neratinib in a dose-dependent manner at 10^4 cells/well. 24 hours later, Celltiter-Glo® reagent was added to each well and luminesence intensity was detected on plate reader. Data show means±SD from 3 independent experiments (n=3). *$p<0.01$ neratinib compared to vehicle treated cells. Related to Figure 2.

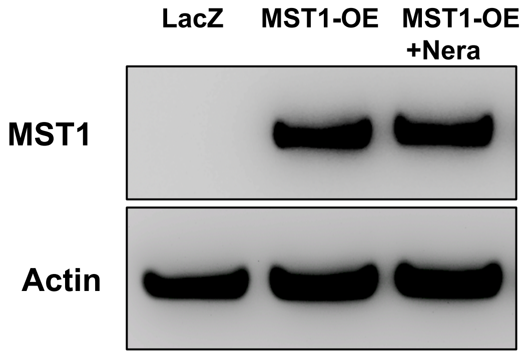

Supplementary Figure 4. MST1 overexpression in human islets. Human islets were infected with Ad-LacZ (control) or Ad-MST1 adenoviruses and exposed to 10 μM neratinib for 48h; successful MST1 overexpression is shown by western blot analysis. Related to Figure 3.

a

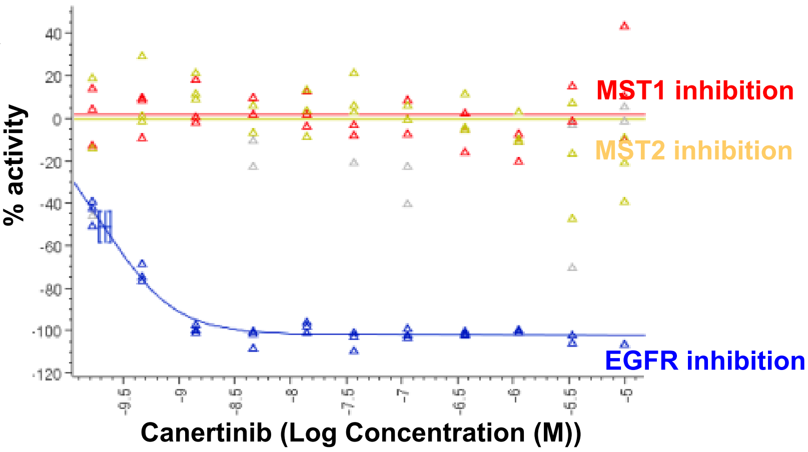

b

	EGFR (IC_{50})	MST1 (IC_{50})	MST2 (IC_{50})
**Canertinib (nM)**	0.21	> 10,000	> 10,000

Supplementary Figure 5. Canertinib's effect on EGFR, MST1 and MST2 inhibition. Biochemical kinase assays in dose response to EGFR (blue), MST1 (red) and MST2 (yellow) inhibition by canertinib **(A)** and IC_{50} summary table **(B).** Data show means from 3 independent experiments (n=3). Related to Figure 5.

a

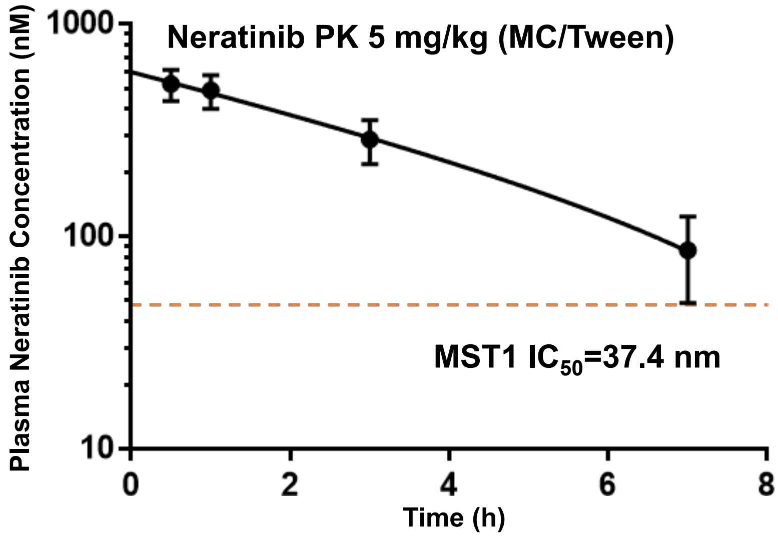

Animal	$t_{1/2}$	C_{max}	T_{max}	AUC_{0-24}	AUC_{LAST}	AUC_{INF}	Cl	MRT	V_D
	(hr)	nM	(hr)	(hr*nM)	(hr*nM)	(hr*nM)	(mL/min/kg)	(hr)	(L/kg)
**1**	4.0	468.6	0.5	1621.8	1621.8	2364.4	253.1	2.7	86.9
**2**	1.9	556.6	1.0	1967.1	1967.1	2153.7	277.9	2.3	46.7
**3**	1.9	623.0	0.5	2125.2	2125.2	2298.5	260.4	2.3	43.1
AVERAGE	2.6	549.4	0.7	1904.7	1904.7	2272.2	263.8	2.4	58.9
ST DEV	1.2	77.4	0.3	257.4	257.4	107.8	12.7	0.2	24.3
CV%	0.5	0.1	0.4	0.1	0.1	0.0	0.0	0.1	0.4

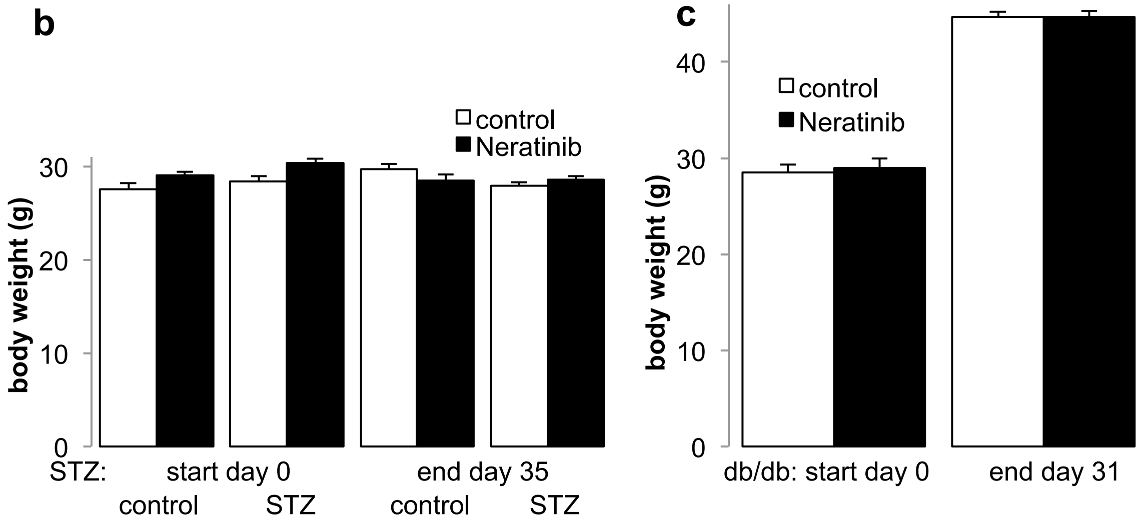

Supplementary Figure 6. (a) Neratinib's IP PK profile in mice and body weights. Neratinib was dosed at 5 mg/kg IP to mice (n=3) which were fasted overnight. The compound was administered in 30% PEG400: 0.5% Tween80: 5% propylene glycol in saline through a single dose. Plasma samples were collected at 30 min., 1, 3 and 7 h post dosing and analyzed by LC-MS to determine the plasma Neratinib concentration. **(b,c)** Mean body weights of all mice in the study from the MLD-STZ mice and db/db mice. Related to Figures 6 and 7.

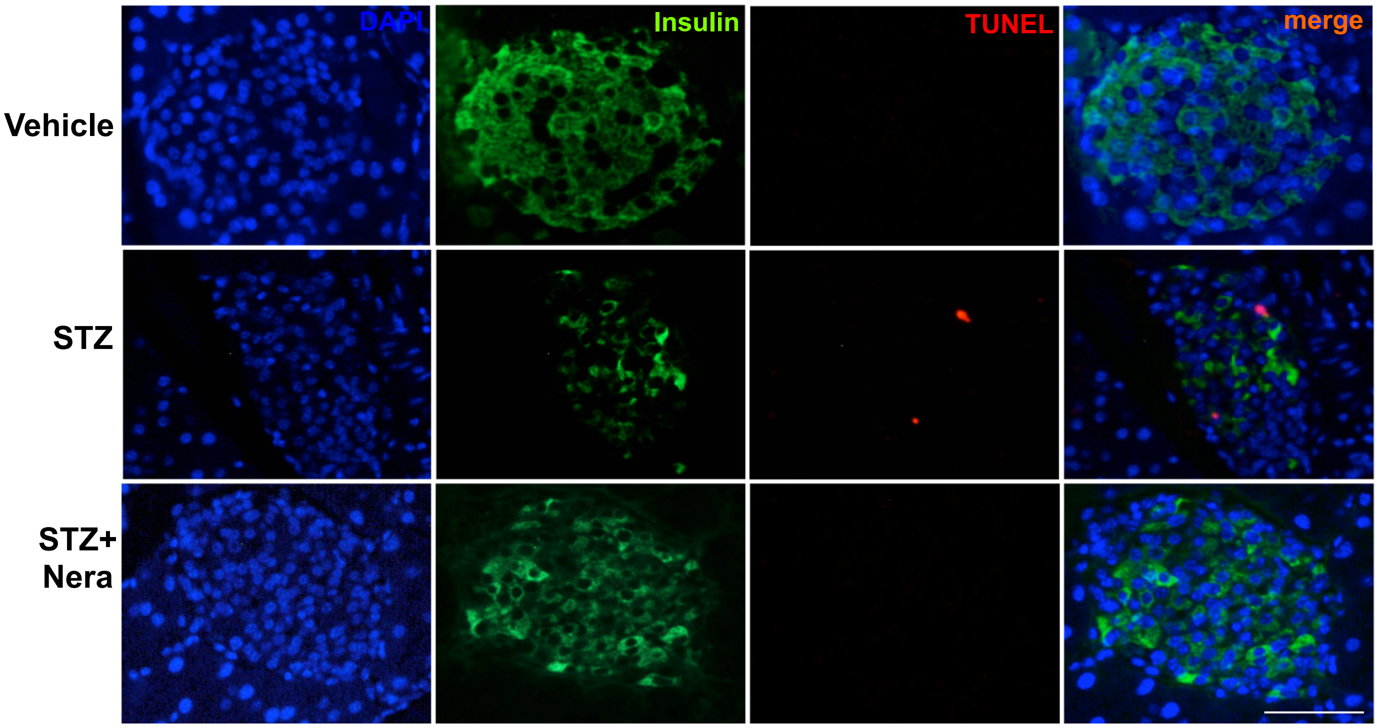

Supplementary Figure 7. Neratinib restores β-cell survival in MLD-STZ-induced diabetes. Representative triple-stainings of pancreatic sections for TUNEL (red), insulin (green) and DAPI (blue) shown from MLD-STZ diabetic mice (STZ) treated with neratinib or vehicle throughout the whole experiment of 35 days and their control (vehicle). Quantification of data are shown in Figure 6h. Related to Figure 6. Scale bar, 100 μm.

Supplementary Figure 8

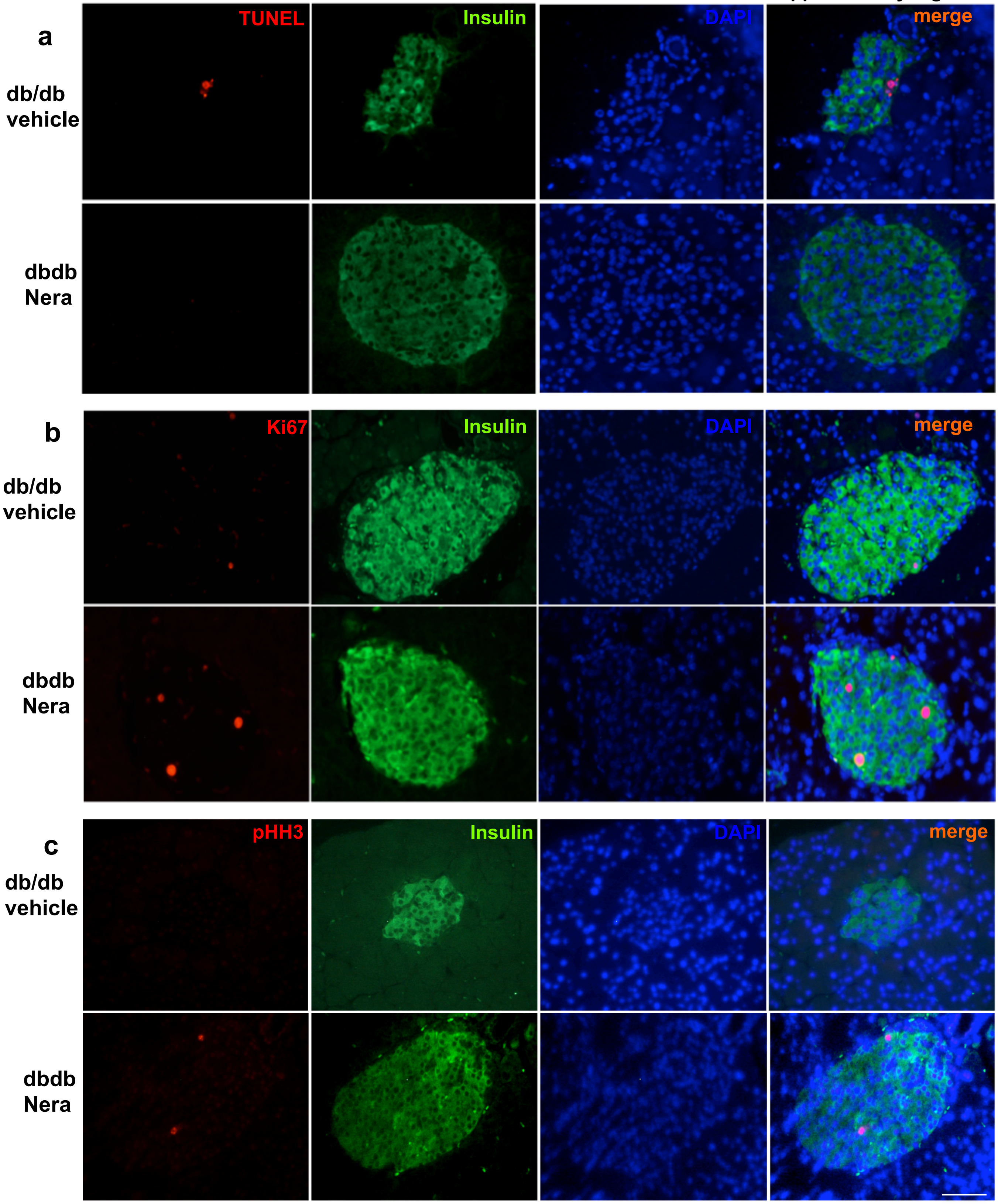

Supplementary Figure 8. Neratinib restores β-cell survival in db/db mice. Representative triple-stainings of pancreatic sections for TUNEL (a), Ki67 (b) and pHH3 (c) (red), insulin (green) and DAPI (blue) shown from db/db diabetic mice (dbdb) treated with neratinib or vehicle throughout the whole experiment of 31 days. Quantification of data are shown in Figure 8h-j. Related to Figure 8. Scale bar, 100 µm.

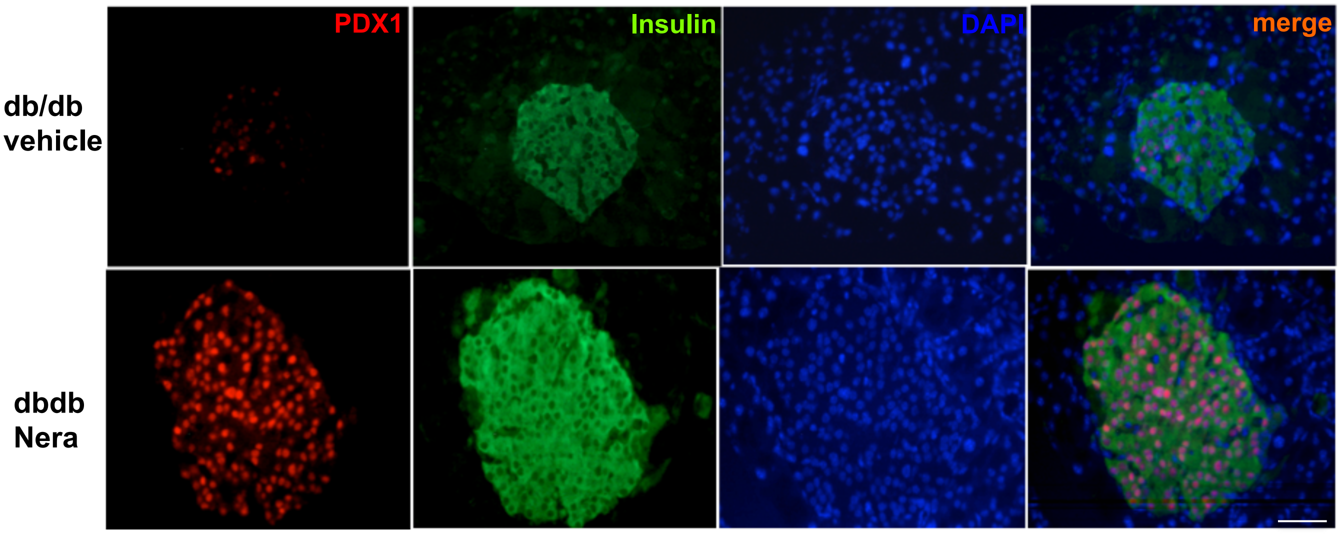

Supplementary Figure 9. Neratinib restores PDX1 in db/db mice. Representative triple-stainings of pancreatic sections for PDX1 (red), insulin (green) and DAPI (blue) shown from db/db diabetic mice (dbdb) treated with neratinib or vehicle throughout the whole experiment of 31 days. Quantification of data are shown in Figure 8k. Related to Figure 8. Scale bar, 100 μm.

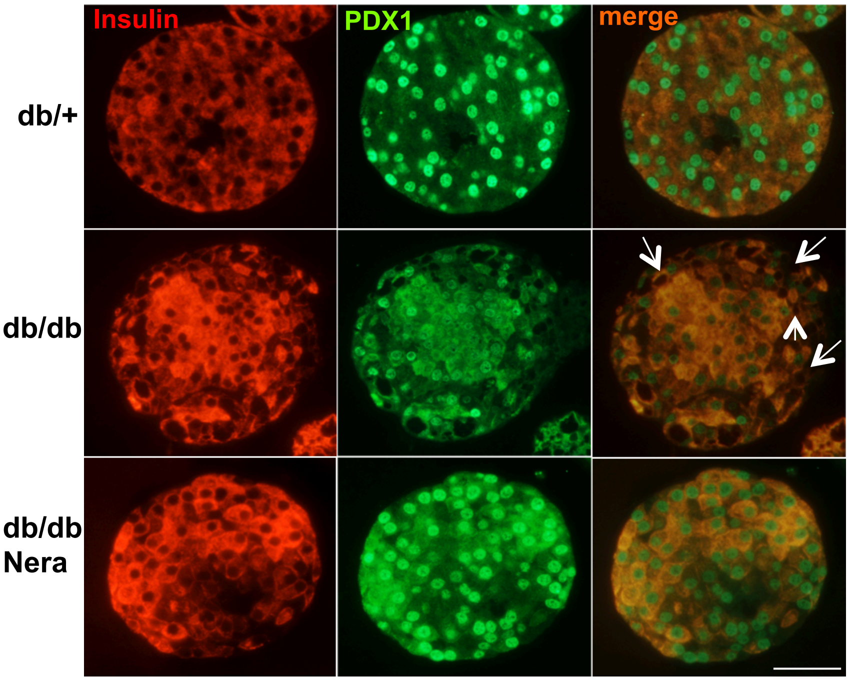

Supplementary Figure 10. Neratinib improves PDX1 nuclear localization in db/db mouse islets ex vivo. Representative double-stainings for PDX1 (green) and insulin (red) shown from isolated islets from 10-week old heterozygous db/+ mice or db/db littermates exposed to vehicle or to 10 μM neratinib for 24h. Representative microscopical images are shown. White arrow point to PDX1 deficient nuclei in db/db mouse islets. Related to Figure 9. Scale bar, 100 μm

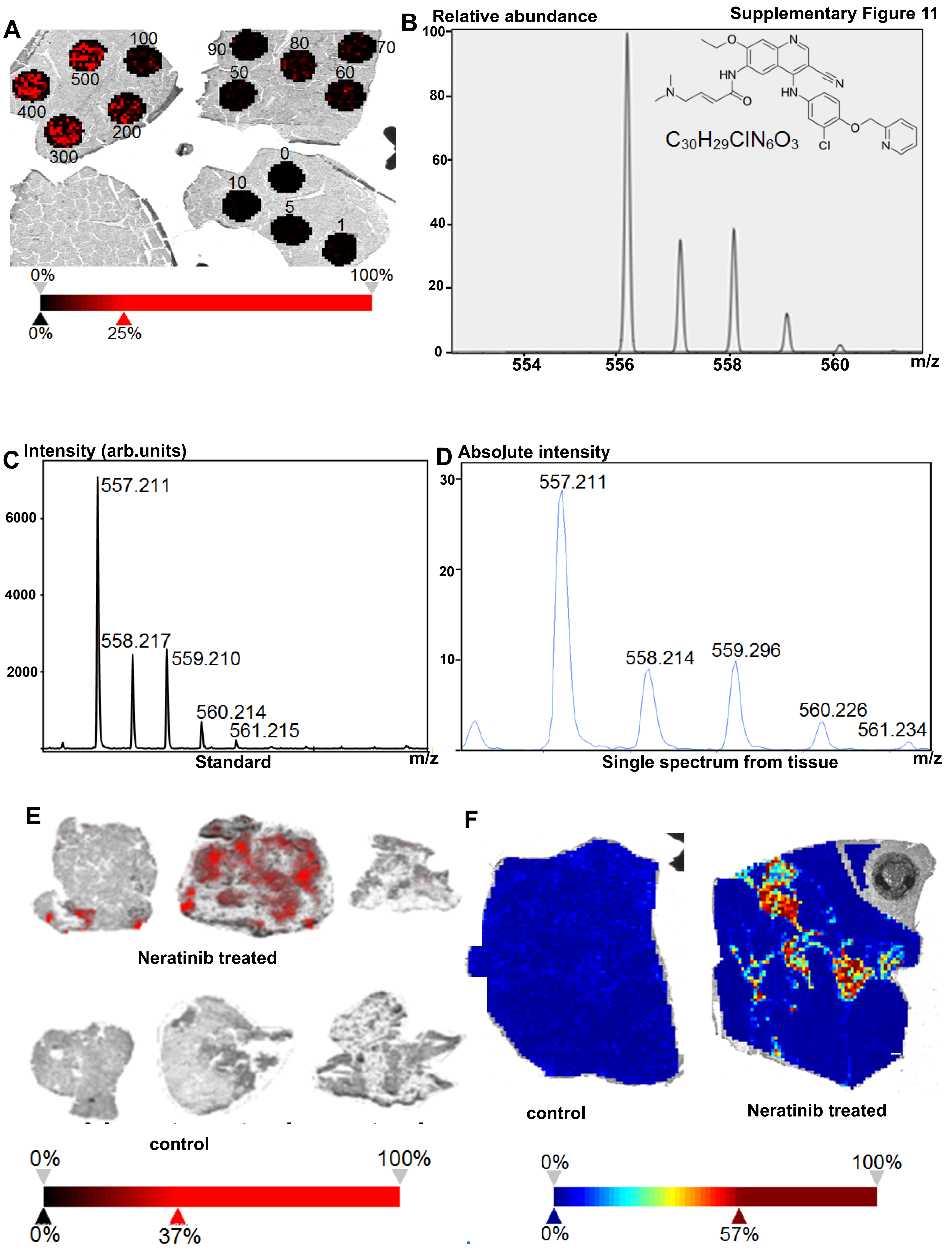
Supplementary Figure 11
A
100
90
80
70
500
50
60
400
200
300
0
10
5
1
0%
100%
0%
25%
B
Relative abundance
100
80
60
40
20
0
554
556
558
560
m/z
$C_{30}H_{29}ClN_6O_3$
C
Intensity (arb.units)
6000
4000
2000
557.211
558.217
559.210
560.214
561.215
Standard
m/z
D
Absolute intensity
30
20
10
557.211
558.214
559.296
560.226
561.234
Single spectrum from tissue
m/z
E
Neratinib treated
control
0%
100%
0%
37%
F
control
Neratinib treated
0%
100%
0%
57%

Supplementary Figure 11. Neratinib is enriched and distributed throughout the pancreas. MALDI Imaging MS of Neratinib for localization of drug distribution in mice tissue sections. **(A)** The dynamic range of the Neratinib signal (monoisotopic peak) in mice liver tissue sections after spotting neratinib standard with concentrations ranging from 0 to 500 pmol/µl is presented. **(B)** The single spectrum of the Neratinib standard spotted on a MALDI steel target shows the distinct isotopic pattern of the drug **(C)** compared to the simulated isotope pattern of $C_{30}H_{29}ClN_6O_3$ (Patiny and Borel, 2013). **(D)** This pattern could be unambiguously detected in the MALDI imaging MS study of pancreas tissue sections as shown by the representative single spectrum. **(E)** The neratinib signal at m/z 577.2 is clearly located in the treated pancreas sections (top) and absent in the non-treated control sections from obese db/db mice used in the study (bottom). **(F)** Distribution of Neratinib in the pancreas sections of wild-type mice 4h after injection (right) compared to the non-treated control (left). Scale bar, 100 µm
