# Supplementary material for: Neratinib protects pancreatic beta cells in diabetes
Source: Nat Commun. 2019 Nov 1;10:5015. doi: 10.1038/s41467-019-12880-5 (PMC6825211; doi:10.1038/s41467-019-12880-5)
Supplement: Supplementary file 2 — Reporting Summary [file 41467_2019_12880_MOESM2_ESM.pdf]

nature research

Corresponding author(s): Matthew S. Tremblay, Weijun Shen and Kathrin Maedler

Last updated by author(s): Jun 7, 2019

Reporting Summary

Nature Research wishes to improve the reproducibility of the work that we publish. This form provides structure for consistency and transparency in reporting. For further information on Nature Research policies, see Authors & Referees and the Editorial Policy Checklist.

Statistics

For all statistical analyses, confirm that the following items are present in the figure legend, table legend, main text, or Methods section.

n/a	Confirmed	
☐	☒	The exact sample size (n) for each experimental group/condition, given as a discrete number and unit of measurement
☐	☒	A statement on whether measurements were taken from distinct samples or whether the same sample was measured repeatedly
☐	☒	The statistical test(s) used AND whether they are one- or two-sided<br>*Only common tests should be described solely by name; describe more complex techniques in the Methods section.*
☒	☐	A description of all covariates tested
☐	☒	A description of any assumptions or corrections, such as tests of normality and adjustment for multiple comparisons
☐	☒	A full description of the statistical parameters including central tendency (e.g. means) or other basic estimates (e.g. regression coefficient) AND variation (e.g. standard deviation) or associated estimates of uncertainty (e.g. confidence intervals)
☐	☒	For null hypothesis testing, the test statistic (e.g. F, t, r) with confidence intervals, effect sizes, degrees of freedom and P value noted<br>*Give P values as exact values whenever suitable.*
☒	☐	For Bayesian analysis, information on the choice of priors and Markov chain Monte Carlo settings
☒	☐	For hierarchical and complex designs, identification of the appropriate level for tests and full reporting of outcomes
☒	☐	Estimates of effect sizes (e.g. Cohen's d, Pearson's r), indicating how they were calculated

Our web collection on statistics for biologists contains articles on many of the points above.

Software and code

Policy information about availability of computer code

Data collection	High throughput screening data were collected on the EnVision Multilabel Plate Reader System from Perkin Elmer. Nanosyn kinase assay plates were run on LabChip 3000/Caliper. Immunostainings were analyzed with a Nikon MEA53200 microscope and images were acquired using NIS-Elements software (Nikon). Western Blot images were collected using DocIT®LS image acquisition 6.6a (UVP). The qPCR reaction was performed and analyzed by the Applied Biosystems ViiA 7 real-time PCR system. MALDI spectra were recorded using a Bruker autofleX speed mass spectrometer (Bruker Daltonics).
Data analysis	High throughput screening data was analyzed with Genedata Screener from Genedata. Nanosyn kinase assay was analysed on a HTS Well Analyzer and XLfit to fit the curve. Immunostainings were analysed and quantified using NIS-Elements software (Nikon). Western Blots were quantified using DocIT®LS image acquisition 6.6a (UVP). MALDI unprocessed raw data were imported and analyzed by the Software SCiLS Lab, version 2016b (SCiLS GmbH, Bremen, Germany).

For manuscripts utilizing custom algorithms or software that are central to the research but not yet described in published literature, software must be made available to editors/reviewers. We strongly encourage code deposition in a community repository (e.g. GitHub). See the Nature Research guidelines for submitting code & software for further information.

Data

Policy information about availability of data

All manuscripts must include a data availability statement. This statement should provide the following information, where applicable:

- Accession codes, unique identifiers, or web links for publicly available datasets
- A list of figures that have associated raw data
- A description of any restrictions on data availability

All data generated or analyzed during this study are included in this published article and its supplementary information files. All other data are available from the corresponding authors upon reasonable request.

Field-specific reporting

Please select the one below that is the best fit for your research. If you are not sure, read the appropriate sections before making your selection.

☒ Life sciences ☐ Behavioural & social sciences ☐ Ecological, evolutionary & environmental sciences

For a reference copy of the document with all sections, see nature.com/documents/nr-reporting-summary-flat.pdf

Life sciences study design

All studies must disclose on these points even when the disclosure is negative.

Sample size	Sample size was selected based on our previous studies and the similar work reported in the literature to ensure adequate reproducibility of data. For human islet experiments: at least 3 independent experiments from 3 independent organ donors were used. For mouse islets and INS1 cells: minimum 3 individual mice and at least 3 independent cultures (cell lines). Other specimen: at least 3 independent samples/ mice Sample size is reported in all figure legends.
Data exclusions	In one single experiment, one single mouse (#1 out of 10) was excluded from the STZ-injected group (random glucose measures, ipGTT), as this mouse did not respond to the STZ injection.
Replication	All attempts for replication were successful. All experimental findings were reproduced as stated in figure legends.
Randomization	Animals were randomly assigned to the treatment conditions by the animal technician before the experiment started. She was not involved in the experiment thereafter. Body weight and glucose measurements were performed before the start of the treatment to ensure equal distribution among the groups. For all cell culture experiments, samples were randomly assigned for control or treatment.
Blinding	All analysis of apoptosis (TUNEL staining), proliferation (Ki67 and pHH3 staining), and beta-cell mass, in human and mouse islets and in mouse pancreata (Fig. 3C,D; 4D-G; 6G-I; 7G-J; 8A-B and 8E-F) were done fully blinded. Labeling of sections was done by numbering at the time of sectioning. Data were analyzed in a randomized manner by two or more investigators, all blinded to the treatment conditions.

Reporting for specific materials, systems and methods

We require information from authors about some types of materials, experimental systems and methods used in many studies. Here, indicate whether each material, system or method listed is relevant to your study. If you are not sure if a list item applies to your research, read the appropriate section before selecting a response.

Materials & experimental systems

n/a	Involved in the study	
☐	☒	Antibodies
☐	☒	Eukaryotic cell lines
☐	☐	Palaeontology
☐	☒	Animals and other organisms
☒	☐	Human research participants
☒	☐	Clinical data

Methods

n/a	Involved in the study	
☒	☐	ChIP-seq
☒	☐	Flow cytometry
☒	☐	MRI-based neuroimaging

Antibodies

Antibodies used	Immunohistochemistry: anti-PDX-1 (Abcam; 47267), anti-Glut2 (Chemicon; 07-1402), anti-Ki67 (Dako; M7249), anti-phospho-Histone H3 (Ser10; Merck 06-570) and anti-NKX6.1 (DSHB, University of Iowa #F55A12) in combination with TSA (Invitrogen #T30955).<br>Western Blotting: rabbit anti-cleaved caspase-3 (#9664), rabbit anti-PARP (#9532), rabbit anti-cleaved PARP (rat specific #9545), rabbit anti-phospho YAP(S127) (#4911), rabbit anti-LATS2 (#5888), rabbit anti-tubulin (#2146), rabbit anti-GAPDH (#2118), rabbit anti-β-actin (#4967) (all Cell Signaling Technology) and rabbit anti-PDX1 (#47267) and rabbit anti-p-MST1 (#79199) (both from Abcam).
Validation	All antibodies used in this study are commercially available and were used according to the manufacturer, who has validated the antibodies before.

Eukaryotic cell lines

Policy information about cell lines

Cell line source(s)	INS-1E cells were used, which are a well reported and well studied model for pancreatic beta-cells. INS-1E were originally provided by Claes Wollheim, Geneva. Well documented mouse RAW 264.7 cells (ATCC® TIB-71™) were purchased from ATCC.

Authentication	INS-1E cells secreted insulin and all tests for beta-cell specific transcription factors were positive, as tested and reported earlier by us (Oncotarget. 2016 Aug 2;7(31):48963-48977; Nat Med. 2014 Apr;20(4):385-397).
Mycoplasma contamination	We routinely test all cell lines for mycoplasma. Tests were negative.
Commonly misidentified lines (See ICLAC register)	No commonly misidentified lines were used.

Palaeontology

Specimen provenance	*Provide provenance information for specimens and describe permits that were obtained for the work (including the name of the issuing authority, the date of issue, and any identifying information).*
Specimen deposition	*Indicate where the specimens have been deposited to permit free access by other researchers.*
Dating methods	*If new dates are provided, describe how they were obtained (e.g. collection, storage, sample pretreatment and measurement), where they were obtained (i.e. lab name), the calibration program and the protocol for quality assurance OR state that no new dates are provided.*

☐ Tick this box to confirm that the raw and calibrated dates are available in the paper or in Supplementary Information.

Animals and other organisms

Policy information about studies involving animals; ARRIVE guidelines recommended for reporting animal research

Laboratory animals	Leptin db/db mice (cat. # 000642) were purchased from Jackson Laboratories (Bar Harbor, ME). C57BL/6J mice were originally purchased from Jackson Laboratories (Bar Harbor, ME) and bred in our mouse facility. Original breeders are replaced each year. Only male mice were used for studies.
Wild animals	This study did not involve wild animals.
Field-collected samples	This study did not involve field-collected samples
Ethics oversight	All animal protocols were approved by the Bremen Senate (Senator for Science, Health and consumer protection). Ethical approval for the use of human islets had been granted by the Ethics Committee of the University of Bremen.

Note that full information on the approval of the study protocol must also be provided in the manuscript.
